# (-)-Shikimic Acid as a Chiral Building Block for the Synthesis of New Cytotoxic 6-Aza-Analogues of Angucyclinones

**DOI:** 10.3390/molecules23061422

**Published:** 2018-06-12

**Authors:** Natalia Quiñones, Santiago Hernández, Luis Espinoza Catalán, Joan Villena, Ivan Brito, Alan R. Cabrera, Cristian O. Salas, Mauricio A. Cuellar

**Affiliations:** 1 Centro de Investigación Farmacopea Chilena, Escuela de Química y Farmacia, Facultad de Farmacia, Universidad de Valparaíso, Av. Gran Bretaña N° 1093, Valparaíso 2340000, Chile; natalia.quinones@uv.cl (N.Q.); santiago.hernandez@postgrado.uv.cl (S.H.); 2 Departamento de Química, Universidad Técnica Federico Santa María, Av. España N° 1680, Valparaíso 2340000, Chile; luis.espinozac@usm.cl; 3 Centro de Investigaciones Biomédicas, Escuela de Medicina, Universidad de Valparaíso, Av. Hontaneda N° 2664, Valparaíso 2340000, Chile; juan.villena@uv.cl; 4 Departamento de Química, Facultad de Ciencias Básicas, Universidad de Antofagasta, Av. Angamos 601, Antofagasta 1240000, Chile; ivan.brito@uantof.cl; 5 Departamento de Química Orgánica, Facultad de Química, Pontificia Universidad Católica de Chile, Vicuña Mackenna N° 4860, Santiago 6094411, Chile; arcabrer@uc.cl

**Keywords:** (-)-Shikimic acid, angucyclinone derivatives, hetero-Diels-Alder, cytotoxicity, cancer cell lines

## Abstract

We describe the syntheses of nine new angucyclinone 6-aza-analogues, achieved through a hetero Diels-Alder reaction between the shikimic acid derivative-azadiene **13**, with different naphthoquinones. The cytotoxic activity of the new synthesized compounds and five angucyclinones, previously reported, was evaluated in vitro against three cancer cell lines: PC-3 (prostate cancer), HT-29 (colon cancer), MCF-7 (breast cancer), and one non-tumoral cell line, human colon epithelial cells (CCD841 CoN). Our results showed that most 6-azadiene derivatives exhibited significant cytotoxic activities, which was demonstrated by their IC_50_ values (less than 10 μM), especially for the most sensitive cells, PC-3 and HT-29. From a chemical point of view, depending on the protected group of ring A and the pattern of substitution on ring D, cytotoxicity elicited these compounds, in terms of their potency and selectivity. Therefore, according to these chemical features, the most promising agents for every cancer cell line were **7a**, **17,** and **19c** for PC-3 cells; **7a**, **17,** and **20** for HT-29 cells, and **19a** for MCF-7 cells.

## 1. Introduction

**Figure 1:**
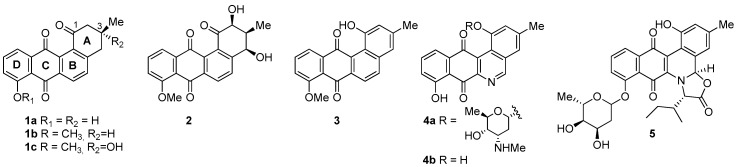
Some examples of natural angucyclinones.

Among the family of polycyclic quinones, angucyclines, and their respective aglycones (angucyclinones), are interesting compounds due to their chemical structural variety and their biological properties [1]. Both compounds correspond to secondary metabolites from numerous microorganisms that belong to spore forming actinomycetes [2]. This group of metabolites exhibit significant biological activities [3,4,5,6], which are not restricted to any particular type of action. However, it is noted that some angucyclines and angucyclinones elicit interesting antitumor activity, as well as acting as hydroxylase and/or mono-oxygenase inhibitors, potent inhibitors of blood platelet aggregation, and exhibit antibacterial or antiviral activity [3,4,5,6]. From a chemical viewpoint, angucyclines belong to the polyketide class of secondary metabolites where their structures are derived from hypothetical decaketides, which are built up in a biosynthetic pathway that involve one acetylCoA, nine malonyl-CoA units, and the Type II polyketide synthase enzyme [3]. These poliketides are transformed in the respective benz[*a*]anthracene backbone, which is a tetracyclic system intermediary that will later be used to obtain several angucyline/angucyclinone-derived molecules. Likewise, angucyclinones have several substitution patterns on their tetracyclic scaffold (Figure 1) [7,8], with the most important and common being the oxygenated moiety on C-1 and the stereogenic centre on C-3, as shown in ochromycinone (**1a**) [9], rubiginone B2 (**1b**) [10], and tetrangomycin (**1c**) [11]. In the ring-A, a highly functionalised system is observed in compounds as (+)-rubiginone D2 (**2**) [12] or the aromatization of this ring exhibits these kind of compounds (**3**) [13]. The first natural 6-aza-analogues of angucyclinones reported were benzo[*b*]phenanthridine derivatives, such as phenanthroviridin (**4a**) and an aglycon phenanthroviridone (**4b**) from *Streptomyces murayamaensis* [14]. Both compounds are active against the lung carcinoma, MBA9812, in mice [15]. Additionally, phenanthroviridone (**4b)** has antibacterial activities against *Staphylococcus aureus* [16] and shows significant in vitro cytotoxicity toward SF-268 (IC_50_ = 0.09 μM) and MCF-7 (IC_50_ = 0.17 μM) [17]. In addition, other 6-aza-analogues of angucyclinones that have been thoroughly studied correspond to jadomycins, which contain an amino acid fragment as part of the phenanthridine scaffold. An example of these compounds is jadomycin B (**5**) (Figure 1), which also exhibits interesting antibacterial or cytotoxic properties [18,19,20,21]. Within the research on the development of new cytotoxic analogues, Yang et al. reported a series of new derivatives using several types of natural amino acids and studied their cytotoxic activity against the tumour cells, MCF-7 and HCT116 [22]. Their results indicated that these compounds elicit moderate activity on these cells (1.0–10 μM). Rohr published a comprehensive review on the structure and biological properties of these compounds [23].

**Figure 2:**
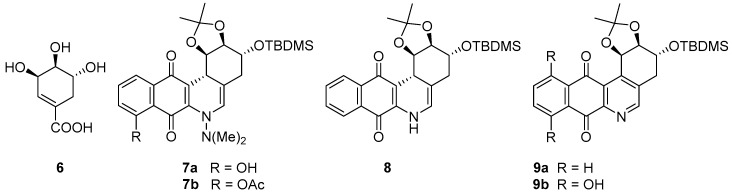
Chemical structures of (-)-shikimic acid and 6-aza-angucyclinone derivatives.

Several synthetic strategies have been considered to access these angular tetracyclic systems [23,24,25,26]. However, the most effective have been the Diels–Alder cycloadditions, which have successfully furnished some angucycline/angucyclinone antibiotics and their analogues [8,27,28]. Considering this pivotal reaction in the synthetic route, azadiene derivatives are important reagents that have led to the incorporation of a nitrogen ring in the ring-B of these tetracyclic systems [29,30,31]. In this respect, we have reported preliminary studies on the synthesis of 6-aza-analogues of angucyclinones (**7**–**9**), starting from (-)-shikimic acid (**6**) (Figure 2) [32].

Despite this chemical and biological diversity of angucyclinone derivatives, none of these compounds have been developed into clinically applicable drugs, often due to toxicity or solubility issues [23]. Hence, the search for new derivatives, with more promising potency and selectivity, has led to research about the design and synthesis of structural analogues or isosters of angucyclinones [33]. Valderrama et al. studied the synthesis and anticancer activity of angucyclinone 5-aza analogues, with the goal of establishing structure-activity relationships (SAR) [34]. In this sense, we aim to contribute to these state-of-the-art compounds by first developing 6-aza-analogues of angucyclinones, shown in Figure 2 [32], which are closely related to the aforementioned natural products. Therefore, in this study, we reported on the synthesis of new analogues of **9**–**10** and delineate the importance of the (-)-shikimic-acid framework on ring A on the cytotoxic properties for all these compounds.

## 2. Results and Discussion

### 2.1. Synthesis

**Scheme 1:**
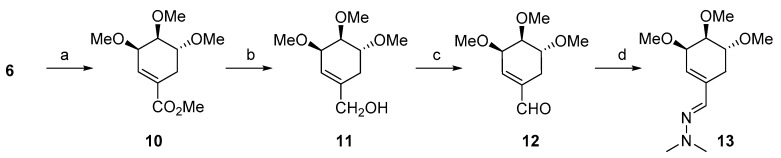
*Conditions and reagents*: (a) REF [35]; (b) DIBAL-H, toluene, −78 °C, 2 h (92%); (c) PCC, CH_2_Cl_2_, r.t., 1 h (51%); and (d) NH_2_NMe_2_, MgSO_4_, CH_2_Cl_2_, r.t., 24 h (42%).

For the synthesis of these new 6-aza-angucycline derivatives, the Diels-Alder cycloaddition was the key step. Because of this, the required azadiene, **13** was obtained from the (-)-shikimic acid, according to the synthetic pathway shown in Scheme 1. Firstly, the (-)-shikimic acid (**6**) was transformed to **10** using reaction conditions reported by Jeso et al. [35]. Later, **10** was treated with diisobutylaluminum hydride solution (DIBAL-H) in toluene at −78 °C and gave the corresponding allylic alcohol, **11**, in a 92% yield. Oxidation of **11** with pyridinium chlorochromate (PCC) in CH_2_Cl_2_ afforded a 51% yield of the aldehyde **12**, which, upon treatment with *N*,*N*-dimethylhydrazine in CH_2_Cl_2_, gave the corresponding hydrazine, **13** (42%). 

**Scheme 2:**
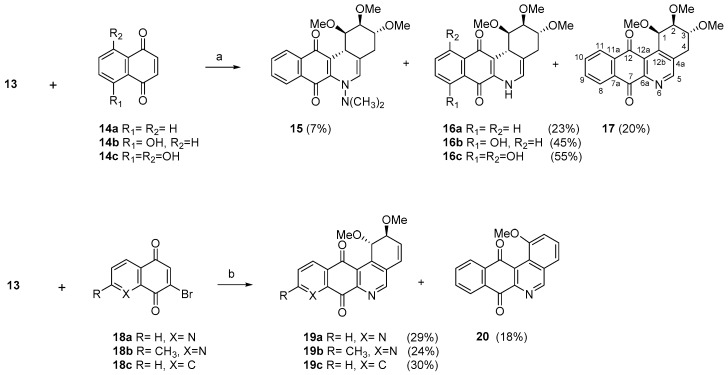
Reagents and conditions: (a): MeCN, reflux, 3 d; (b) NaHCO_3_, MeCN, reflux, 5 h.

To carry out the Diels-Alder reaction (Scheme 2), the 1,4-naphtoquinones derivatives were commercial (**14a**–**c** and **18c**) or synthetic compounds (**18a**–**b** [36]), which were condensed with azadiene **13** in MeCN under thermal activation. When the simplest naphthoquinone was used (**14a**), three compounds of this cycloaddition were obtained (**15**, **16a**, and **17**), which corresponded sequentially to the initial adduct until the aromatization product. However, when mono or di-hydroxyl-naphthoquinones were used (**14b** and **14c**), only the enamine derivatives were obtained (**16b**–**c**), with a good yield (45–55%).

**Figure 3:**
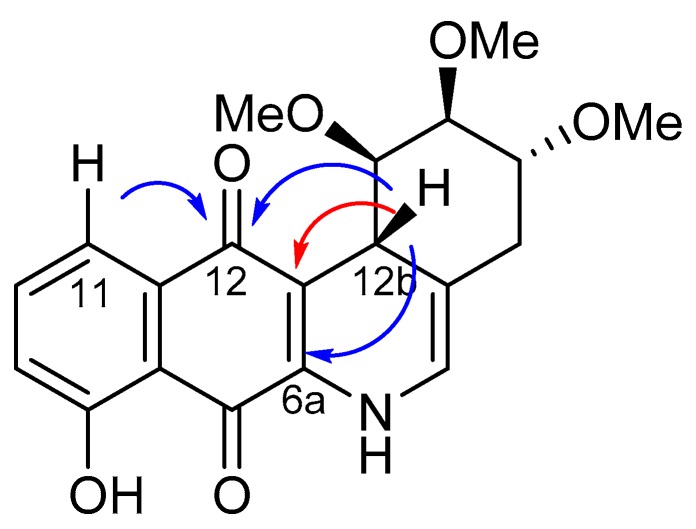
Major HMBC correlations observed for compound **16b**. The ^3^*J*_HC_ and ^2^*J*_HC_ correlations are shown in blue and red, respectively.

Structural confirmation of all new compounds was based on a careful analysis and extensive use of ^1^H and ^13^C NMR spectra, with the help of a combination of 1D and 2D experiments, especially, including heteronuclear multiple-bond correlations (HMBC and HSQC). Specifically, the configuration of the stereogenic centre at the C-12b position of the enamine derivatives, **15** and **16a**–**c**, were established from the presence in its ^1^H NMR spectrums of a doublet, at around δ 4.2 ppm, due to its H-12b proton, which is coupled with the H-1 proton with a coupling constant of nearly 10 Hz [32]. For example, a full analysis of the structural determination of the compound, **16b,** in the ^1^H NMR spectrum, showed signals at δH = 4.26 ppm (1H, d, *J* = 10.0 Hz) were assigned to hydrogen H-12b, which showed an HSQC ^1^*J*_HC_ correlation with C-12b at δC = 34.7 ppm and an HMBC ^3^*J*_HC_ correlation with carbonyl carbon C-12 at δC = 182.2 ppm. Additionally, H-12b showed HMBC correlations with quaternary aromatic carbon C-6a (δC = 139.6 ppm; ^3^*J*_HC_) and with C-12a (δC = 115.9 ppm; ^2^*J*_HC_) (Figure 3). On the other hand, the signal at δH = 7.62 ppm (H-11) showed an HSQC correlation with C-11 at δC = 118.9 ppm (^1^*J*_HC_) and HMBC correlations with C-12 (δC = 182.2 ppm) and C-9 (δC = 121.9 ppm; ^3^*J*_HC_). All these correlations are shown in Figure 3. In addition, the unequivocal structure of **16b** was established from the crystallographic data, which is discussed in Section 2.2. 

To obtain 6,8-diaza-anguciclyne derivatives using the same strategy, halogenated-naphthoquinone derivatives (**18a**–**b**) were required to obtain only the desired regioisomer due to the orientation effect of the halogen atom on the quinone [37,38] and to the increase of the reactivity of dienophile. The only change for the condition reactions for this cycloaddition was the addition of NaHCO_3_, which is necessary when a haloquinone is used [37,39]. For these cases, the reaction times were less than the described above, decreasing from three days to five hours. However, unless the obtained products corresponded to an aromatic system, an unexpected chemical modification was observed on ring A. In this ring, which is the shikimic acid-moiety, inversion of the stereochemistry on C-1 and elimination of a methoxy group on C-3 yielded like-**19** products. This behaviour was not observed previously when the azadiene had other protected groups [32]. This effect is probably because methoxy groups are better at leaving a group in this system compared to the ketal or silyl protected groups presented in our first azadiene.

**Scheme 3:**
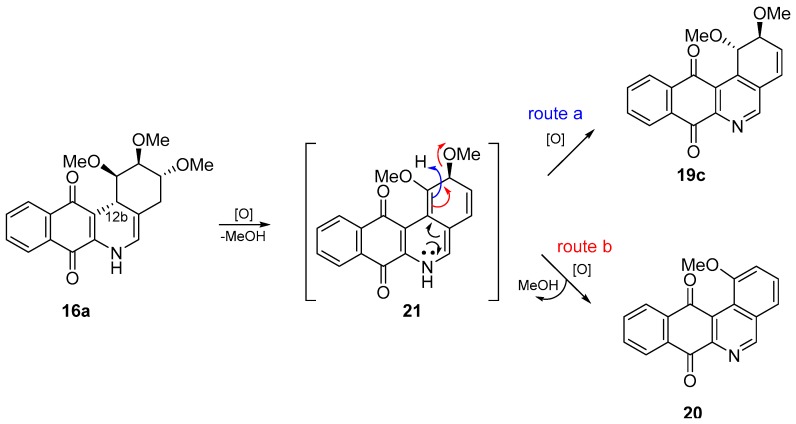
Proposed mechanism for the formation of compounds **19c** and **20**.

**Figure 4:**
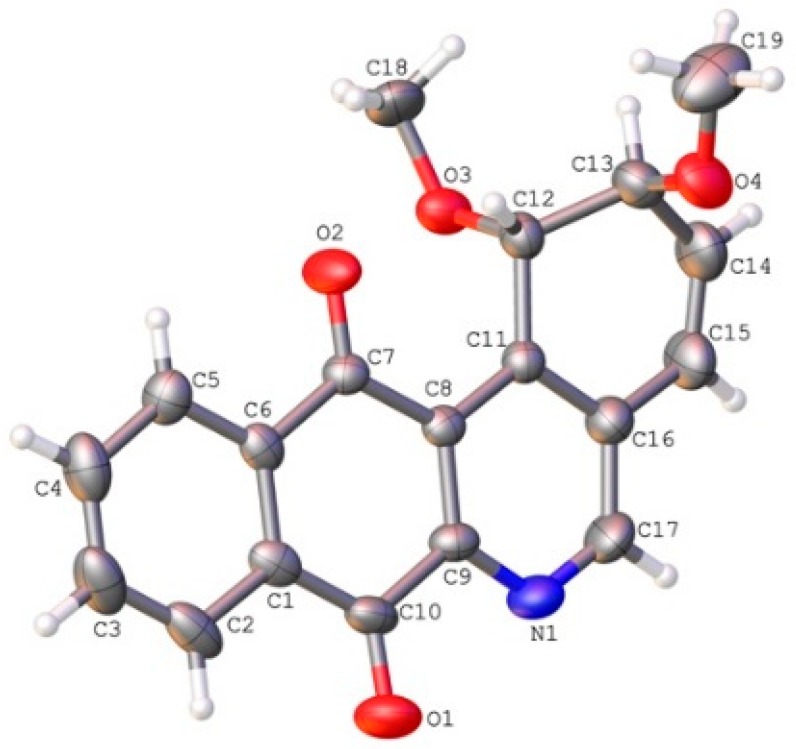
Molecular structure of compound **19c**. Thermal ellipsoids are shown with 30% probability.

To understand the formation of these compounds, we carried out the cycloaddition reaction using 2-bromo-naphthoquinone, **18c**, as starting material, with the goal of analysing the role of the haloquinone. In this case, the same structural product was observed, **19c**, together with a minor full aromatic compound, **20**. The chemical structure of **19c** was unequivocally established from the crystallographic data, as shown in Section 2.2. Considering these results, and other reports that show that when some haloquinones are used in Diels-Alder reaction, several oxidation processes are favourable on the adducts due to their more reactive properties [37]. Based on these antecedents, we proposed an eventual mechanism to explain the formation of **19a**–**c** and **20**, which is outlined in Scheme 3. The formation of **19c** can be accounted for by assuming that, due to the oxidant potential of the haloquinones, the Diels-Alder adduct, **16a**, can be oxidized with additional loss of a methanol in C-3 to obtain the proposed intermediary, **21**. Later, **21** could be transformed via two different pathways. Firstly, **21** is subject to an aromatization process of ring B, resulting in the capture of a hydrogen atom, with an inversion of the configuration on C-1 to obtain compound **19c** (route a, Scheme 3). The change of the configuration on C-1 could be due to that, during this aromatization, the rearrangement from a *cis*-configuration of methoxy groups is favoured on C1-C2 through their more stable *trans*-configuration, as is observed in Figure 4. Secondly, the minor compound, **20**, could be formed by a second elimination of methanol in C-2 during the aromatization process of **21** (route b, Scheme 3).

### 2.2. Crystallographic Studies

The molecular structure of compound **19c** was determined by X-ray monocrystal diffraction (see Figure 4). Compound **19c** exhibited a higher planar fragment between B-C-D heterocyclic fused rings, with a dihedral angle of −175.8(2)° (N1-C9-C10-C1). Ring A showed a deviation from planarity (157.7(0)° C8-C11-C12-C13) and contained two methoxy groups in the C12 and C13 atoms. These substituents present a *trans* configuration among them, regarding the cyclohexane ring, with a dihedral angle of 162.6(0)° (O4-C13-C12-O3). Also, these methoxy groups have a deviation from orthogonality according to the measured dihedral angle (−78.8(1)° C11-C12-C13-O4; −80.7(2)° C8-C11-C12-O3).

### 2.3. Biology

To analyse the scope of the shikimic-fragment incorporated in the angucycline scaffold on the cytotoxic activity of these compounds, we included five previously obtained compounds (Figure 2, [32]). Therefore, the cytotoxicity of the new nine synthesised compounds, and of the five aforementioned compounds, was evaluated in vitro against different cancer cell lines: PC-3 (prostate cancer), HT-29 (colon cancer), MCF-7 (breast cancer), and one non-tumoral cell line, human colon epithelial cells (CCD841 CoN). A conventional colorimetric assay was set up to estimate the IC_50_ values, which represent the concentration of a drug that is required for 50% inhibition in vitro after 72 h of continuous exposure to the test compounds. Several serial dilutions (from 1.25 to 100 µM) for each sample were evaluated in triplicate.

Table 1 shows the IC_50_ values for the cytotoxicity of the compounds, **7**–**9**, **15**–**17**, and **19**–**20**. In general, the activity of 6-aza-angucycline derivatives was quite heterogeneous (IC_50_ range of 119–0.1 μM against some cancer cell lines). However, in most cases, the potency of all assayed compounds was less than 45 μM, especially for the most sensitive cells: PC-3 and HT-29. However, after further cytotoxicity analysis for each cancer cell line, we conclude that:

(i) For PC-3 cells, compounds **7a**, **19a,** and **19b** were the most potent compounds of all assayed, with IC_50_ values less than 1.0 μM. Nevertheless, among these three, **7a** was more selective (SI value = 3.3). Although **16a**, **17**, **19c,** and **20** were less active than the last-mentioned compounds, they showed the highest selectivity (SI values = 4.9, 3.0, 4.4, and 3.0, respectively).
(ii) For HT-29 cells, once more the compounds **7a**, **19a,** and **19b** were the most potent derivatives (IC_50_ values < 0.4 μM). It should be noted that **7a** elicited the best result in selectivity in respect to all assayed compounds in this study. Nevertheless, **19a** and **19b** showed SI values less than 1.0. In addition, **17** and **20** were also interesting compounds due to their moderate cytotoxicity (IC_50_ values ~ 3.0 μM), as well as their good selectivity (SI values = 3.6 and 6.4, respectively).
(iii) For MCF-7, we observed that **19a** was the best compound in potency (IC_50_ value ~ 10 nM) and selectivity (SI value = 4.0). This is an interesting result because this cell line was less sensitive than the other cell lines assayed.

Summing up the previous results and, considering that, an ideal antitumor drug must be cytotoxic and selective to cancer cells, some newly synthesized angucycline derivatives (**7a**, **16a**, **17**, **19a**, **19c,** and **20**), are very promising for the development of new antitumor agents. This sentence is based on National Cancer Institute (NCI) protocols, which consider active compounds exhibiting IC_50_ values < 10 μM or 15 μM (NCI/NIH, 2014). In addition, our results showed that some compounds elicited interesting SI values (>3.0), which gave us an opportunity to understand the structural requirements for obtaining compounds with selective activity toward cancer cells.

Regarding the IC_50_ values and the chemical structures of synthesised compounds, a consistent structure-activity relationship was not established. Nonetheless, we suggest that, from a chemical point of view, there are interesting structural features worth considering, according to the three fragments of these angucycline derivatives:

(i) Considering the size of the protected groups on ring A, the evidence could indicate that groups that are more voluminous generate an increase in cytotoxic activity. This behaviour is clearly observed in all cancer cell lines, when comparing **8** versus **16a** and **7b** with **15**. The same slight tendency is demonstrated when comparing **7a** with **16b**.
(ii) With respect to ring B, it was not possible to indicate that the cytotoxicity of these angucyclines could be related only to the aromatic features of this ring due to the influence of the protected groups. In fact, for voluminous groups on ring A, the cytotoxicity was reduced in the aromatic system (**8** versus **9**). However, when the protected groups were methoxy, a significant increase of activity (almost tenfold) was elicited when this ring was aromatic (**16a** versus **17**). This effect was observed on the three cancer cell lines.
(iii) Finally, when the benzene ring (ring D) was substituted in C-8 for an electron-donating group (hydroxyl group), this modification generated an increase in the cytotoxic activity. This effect was observed for **7a** when comparing it with its analogous **8** on two cancer cell lines (PC-3 and HT-29 cells). This behaviour was similar for **16a** and its related compound **16b**, but not in all three cancer cell lines. On the other hand, the isosteric replacement of the benzene ring by a pyridine ring led to an increase in the potency of the respective derivatives, which is shown when comparing the IC_50_ values between **19c** and **19a**–**b**. These results are in agreement with the data reported by several other authors [23,34,40,41]. Our results indicate that, on three cell lines, the addition of an extra nitrogen atom in the aromatic ring increases cytotoxic activity.

## 3. Materials and Methods

### 3.1. Chemistry

All reagents were purchased from commercial suppliers and used without further purification. The melting reported were uncorrected and determined by an SMP3 instrument (Stuart-Scientific, now Merck KGaA, Darmstadt, Germany) and are uncorrected. Optical rotations were measured with a sodium lamp (λ = 589 nm, D line) on a Perkin Elmer 241 digital polarimeter (Perkin Elmer, Waltham, MA, USA) equipped with 1 dm cells at the temperature indicated in each case. The IR spectra were recorded as NaCl windows in a FT-IR 4600 Jasco spectrometer (Jasco, Tokyo, Japan) and frequencies are reported in cm^−1^. The ^1^H-, ^13^C-, ^13^C DEPT-135, ^13^C DEPT-90, gs 2D HSQC, and gs 2D HMBC NMR spectra were recorded in CDCl_3_ solutions and are referenced to the residual peaks of CHCl_3_ at δ = 7.26 ppm and δ = 77.00 ppm for ^1^H and ^13^C on an Avance 400 Digital NMR spectrometer (Bruker, Rheinstetten, Germany) operating at 400.1 MHz for ^1^H and 100.6 MHz for ^13^C. Mass spectra were obtained with an Exactive™ Plus Orbitrap mass spectrometer (Thermo Fisher Scientific, Bremen, Germany). Silica gel (Merck 200–300 mesh, Merck, Santiago, Chile) was used for C.C. and silica gel plates HF-254 for TLC. ^1^H and ^13^C NMR spectra, IR spectra and HRMS for all compounds and crystallographic data of **19c**, see the Supplementary Materials.

#### 3.1.1. Synthesis of ((3*R*,4*S*,5*R*)-3,4,5-Trimethoxycyclohex-1-en-1-yl) methanol (**11**)

Compound **10** (1.4 g, 6.08 mmol) was dissolved in anhydrous toluene (30 mL), the solution was stirred at −78 °C under nitrogen, DIBAL-H reagent (10 mL of 1.0 M solution in toluene, 10.0 mmol) was added dropwise, and the resulting solution was allowed to warm to room temperature over the course of 12 h. After this period, NaOH solution (0.5 mL, 15% aq) was added dropwise and then 50 mL of water was added. The mixture was extracted with EtOAc and the extracts were dried over MgSO_4_ concentrated in vacuum. The evaporation residue was purified by column chromatography to give compound **11** (1.13 g 5.59 mmol) as a colourless oil, yield 92%; [α]_D_^25^ −141.2 (CH_2_Cl_2_, c = 1.13); IR (NaCl windows) cm^−1^ = 3446 (ν -OH); 1100 (ν C-O) ^1^H-NMR (400 MHz, CDCl_3_) δ: 5.79 (1H, bs, H-2), 4.00 (2H, s, CH_2_OH), 3.97 (1H, bs, H-3), 3.75 (1H, dd, *J* = 7.6, 5.8 Hz, H-5), 3.49 (3H, s, OCH_3_)*, 3.48–3.46 (1H, m, H-4), 3.44 (3H, s, OCH_3_)*, 3.43 (3H, s, OCH_3_)*, 2.49 (1H, dd, *J* = 17.5, 5.3 Hz, H-6), 2.27 (1H, bs, OH), 2.01 (1H, dd, *J* = 17.5, 6.1 Hz, H-6). (*) interchangeable signals. ^13^C-NMR (100 MHz, CDCl_3_) δ: 139.3 (C-1), 119.4 (C-2), 80.2 (C-4), 75.6 (C-5), 74.2 (C-3), 66.0 (CH_2_OH), 58.4 (OCH_3_)*, 57.7 (OCH_3_)*, 57.4 (OCH_3_)*, 30.2 (C-6). (*) interchangeable signals. HRMS (ES^+^): *m*/*z* calcd for C_10_H_18_O_4_ [M + H]^+^: 203.1283; found 203.1278.

#### 3.1.2. Synthesis of (3*R*,4*S*,5*R*)-3,4,5-Trimethoxycyclohex-1-enecarbaldehyde (**12**)

To a magnetically stirred solution of the alcohol **11** (1.13 g, 5.58 mmol) in CH_2_Cl_2_ (50 mL), pyridinium chlorochromate (14.0 mmol) was added. The brown suspension was stirred for 30 min and EtOAc (50 mL) were added to the reaction mixture and the slurry was filtered through a short pad of silica gel, washing copiously with EtOAc. The filtrate was dried, evaporated, and purified by column chromatography to give compound **12** (0.6 g, 2.8 mmol) as a colourless oil, 51% yield. [α]_D_^25^ −364.0 (CH_2_Cl_2_, c = 0.58); IR (NaCl windows) cm^−1^ = 1684 (ν C=O); 1088 (ν C-O). ^1^H-NMR (400 MHz, CDCl_3_) δ: 9.43 (1H, s, HC=O), 6.66 (1H, bs, H-2), 4.18 (1H, bs, H-3), 3.83–3.80 (1H, m, H-5), 3.76–3.72 (1H, m, H-4), 3.49 (3H, s, 3-OCH_3_), 3.46 (3H, s, 4-OCH_3_), 3.34 (3H, s, 5-OCH_3_), 2.37 (2H, d, *J* = 1.6 Hz, H-6). ^13^C NMR (100 MHz, CDCl_3_) δ: 193.4 (C=O), 145.8 (C-2), 138.8 (C-1), 76.4 (C-4), 75.4 (C-3), 74.6 (C-5), 58.4 (4-OCH_3_), 57.7 (3-OCH_3_), 57.4 (5-OCH_3_), 22.8 (C-6). HRMS (ES^+^): *m*/*z* calcd for C_10_H_16_O_4_ [M + H]^+^: 201.1127; found 201.1122.

#### 3.1.3. Synthesis of (E)-1,1-Dimethyl-2-(((3*R*,4*S*,5*R*)-3,4,5-trimethoxycyclohex-1-en-1-yl)methylene)hydrazine (**13**)

A suspension of aldehyde **12** (0.6 g, 3.00 mmol), *N*,*N*-dimethylhydrazine (0,20 mL, 2,66 mmol), and anhydrous MgSO_4_ (150 mg) in CH_2_Cl_2_ (25 mL) was stirred at room temperature for 24 h. The mixture was filtered and the filtrate was evaporated. The residue was purified by column chromatography to afford hydrazone **13** (0.3 g, 1.26 mmol) as a yellow oil, 42% yield. [α]_D_^25^ −272.3 (CH_2_Cl_2_, c = 0.72); IR (NaCl windows) cm^−1^ = 2828 (ν =CH); 1684 (ν C=C); 1560 (ν C=N); 1093 (ν C-N and ν C-O). ^1^H NMR (400 MHz, CDCl_3_) δ: 6.93 (1H, s, CH=N), 5.84–5.80 (1H, m, H-2), 4.09 (1H, t, *J* = 3.8 Hz, H-3), 3.80 – 3.75 (1H, m, H-5), 3.53–3.49 (1H, m, H-4), 3.50 (3H, s, OCH_3_)*, 3.46 (6H, s, OCH_3_)*, 2.87–2.80 (1H, m, H-6), 2.83 (6H, s, N(CH_3_)_2_), 2.28 (1H, dd, *J* = 18.1, 6.3 Hz, H-6). ^13^C NMR (100 MHz, CDCl_3_) δ: 137.0 (C=N), 134.9 (C-1), 123.6 (C-2), 80.2 (C-4), 75.5 (C-5), 74.4 (C-3), 58.1 (OCH_3_)*, 57.5 (OCH_3_)*, 57.1 (OCH_3_)*, 42.7 (N(CH_3_)_2_), 28.2 (C-6). (*) interchangeable signals. HRMS (ES^+^): *m*/*z* calcd for C_12_H_22_N_2_O_3_ [M + H]^+^: 243.1708; found 243.1703.

#### 3.1.4. General Procedure for the Synthesis of the Target Compounds **15**–**17**

Azadiene **13** (300 mg, 1.24 mmol) and appropriate 1,4-napthoquinone (**14**) (1.90 mmol) in anhydrous MeCN (30 mL) were refluxed for 3 days. The reactions products were filtered, concentrated, and purified by column chromatography.

*(1R,2S,3R,12bR)-6-(Dimethylamino)-1,2,3-trimethoxy-1,2,3,4,6,12b-hexahydrobenzo[b]phenanthridine-7,12-dione* (**15**). Red solid (34.6 mg, 7%); mp 128.5 °C; IR (NaCl windows) cm^−1^ = 2823 (ν =CH); 1670 (ν C=O); 1598 (ν C=C); 1264(ν C-O); 1107 (ν C-N). ^1^H NMR (400 MHz, CDCl_3_) δ: 8.05 (1H, dd, *J* = 7.7, 1.2 Hz, H-11), 7.96 (1H, dd, *J* = 7.2, 1.3 Hz, H-8), 7.67–7.59 (2H, m, H-9 + H-10), 6.00 (1H, d, *J* = 0,7 Hz, H-5), 4.25 (1H, d, *J* = 10.0 Hz, H-12b), 3.73 (1H, m, H-3), 3.66 (1H, dd, *J* = 5.7, 3.0 Hz, H-2), 3.57 (3H, s, 2-OCH_3_), 3.38 (1H, dd, *J* = 10.0, 2.8 Hz, H-1), 3.38 (3H, s, 3-OCH_3_), 3.23 (3H, s, 1-OCH_3_), 2.70 (6H, bs, N(CH_3_)_2_), 2.35 (1H, dd, *J* = 14.0, 1.8 Hz, H-4), 2.23 (1H, dd, *J* = 14.0, 1.8 Hz, H-4). ^13^C NMR (100 MHz, CDCl_3_) δ: 183.4 (C-12), 181.1 (C-7), 146.0 (C-6a), 133.2 (C-9), 133.0^#^ (C-7a), 132.5^#^ (C-11a), 132.2 (C-10), 126.0 (C-8), 125.7 (C-11), 118.6* (C-12a), 118.6* (C-4a), 118.4 (C-5), 85.0 (C-1), 77.7 (C-3), 77.0 (C-2), 59.1 (2-OCH_3_), 58.2 (1-OCH_3_), 56.4 (3-OCH_3_), 44.2 (N(CH_3_)_2_), 34.4 (C-12b), 31.1 (C-4). (*,^#^) interchangeable signals. HRMS (ES^+^): *m*/*z* calcd for C_22_H_26_N_2_O_6_ [M + H]^+^: 399.1920; found 399.1911.

*(1R,2S,3R,12bR)-1,2,3-Trimethoxy-1,2,3,4,6,12b-hexahydrobenzo[b]phenanthridine-7,12-dione* (**16a**). Purple solid (101.4 mg, 23%); mp 161.5 °C; IR (NaCl windows) cm^−1^ = 3307 (ν -NH); 1623 (ν C=O); 1585 (ν C=C). ^1^H NMR (400 MHz, CDCl_3_) δ: 8.12 (1H, d, *J* = 7.5 Hz, H-11), 8.00 (1H, d, *J* = 7.7 Hz, H-8), 7.70 (1H, dd, *J* = 7.7, 7.5 Hz, H-9), 7.60 (1H, t, *J* = 7.5 Hz, H-10), 6.77 (1H, s, H-6), 5.99 (1H, d, *J* = 4.0 Hz, H-5), 4.29 (1H, d, *J* = 10.0 Hz, H-12b), 3.77 (1H, m, H-3), 3.64 (1H, bs, H-2), 3.58 (3H, s, 2-OCH_3_), 3.50 (1H, dd, *J* = 10.0, 2.0 Hz, H-1), 3.38 (3H, s, 3-OCH_3_), 3.26 (3H, s, 1-OCH_3_), 2.35 (1H, dd, *J* = 14.0, 1.8 Hz, H-4), 2.23 (1H, dd, *J* = 14.0, 1.3 Hz, H-4). ^13^C NMR (100 MHz, CDCl_3_) δ: 182.9 (C-12), 180.5 (C-7), 139.8 (C-6a), 134.6 (C-8), 133. 8 (C-7a), 131.8 (C-10), 130.2 (C-11a), 126.4 (C-11), 125.7 (C-9), 118.6 (C-5), 115. 6 (C-4a), 110.5 (C-12a), 83.9 (C-1), 77.7 (C-3), 77.4 (C-2), 59.1 (2-OCH_3_), 58.0 (1-OCH_3_), 56.7 (3-OCH_3_), 34.7 (C-12b), 31.3 (C-4). HRMS (ES^+^): *m*/*z* calcd for C_20_H_21_NO_5_ [M + H]^+^: 356.1498; found 356.1484.

*(1R,2S,3R)-1,2,3-Trimethoxy-1,2,3,4-tetrahydrobenzo[b]phenanthridine-7,12-dione* (**17**). Brown solid (87.6 mg, 20%); mp 184.8 °C; IR (NaCl windows) cm^−1^ = 1687(ν C=O); 1591, 1558(ν C=N). ^1^H NMR (400 MHz, CDCl_3_) δ: 8.83 (1H, s, H-5), 8.34–8.32 (1H, m, H-8), 8.25–8.22 (1H, m, H-11), 7.82–7.80 (2H, m, H-9, H-10), 6.19 (1H, d, *J* = 2.8 Hz, H-1), 4.09–4.01 (1H, m, H-3), 3.62 (3H, s, OCH_3_), 3.59 (1H, m, H-4), 3.56 (3H, s, OCH_3_), 3.50 (3H, s, OCH_3_), 3.45 (1H, dd, *J* = 7.6, 2.9 Hz, H-2), 2.89 (1H, dd, *J* = 17.4, 5.0 Hz, H-4). ^13^C NMR (100 MHz, CDCl_3_) δ: 185.2 (C-7), 181.4 (C-12), 154.0 (C-5), 148.2 (C-6a), 145.2 (C-12b), 137.4 (C-4a), 134.5 (C-9), 134.3 (C-10), 134.1 (C-11a), 132.4 (C-12a), 128.0 (C-7a), 127.3 (C-8), 127.2 (C-11), 83.4 (C-2), 75.3 (C-3), 71.5 (C-1), 59.4 (1-OCH_3_), 58.2 (2-OCH_3_), 57.5 (3-OCH_3_), 32.0 (C-4). HRMS (ES^+^): *m*/*z* calcd for C_20_H_19_NO_5_ [M + H]^+^: 354.1341; found 354.1333.

*(1R,2S,3R,12bR)-8-Hydroxy-1,2,3-trimethoxy-1,2,3,4,6,12b-hexahydrobenzo[b]phenanthridine-7,12-dione* (**16b**). Green solid (207.2 mg, 45%); mp 177.3 °C; IR (NaCl windows) cm^−1^ = 3333 (ν -OH); 1660 (ν C=O); 1593 (ν C=C). ^1^H NMR (400 MHz, CDCl_3_) δ: 11.42 (1H, s, Ar-OH), 7.62 (1H, d, *J* = 7.4 Hz, H-11), 7.57 (1H, dd, *J* = 8.2, 7.4 Hz, H-10); 7.10 (1H, d, *J* = 8.2 Hz, H-9), 6.78 (1H, d, *J* = 3.1 Hz, H-6), 5.98 (1H, d, *J* = 4.2 Hz, H-5), 4.26 (1H, d, *J* = 10.0 Hz, H-12b), 3.77 (1H, s, H-3), 3.63 (1H, bs, H-2), 3.56 (3H, s, 2-OCH_3_), 3.48 (1H, d, *J* = 10.0 Hz, H-1), 3.37 (3H, s, 3-OCH_3_), 3.26 (3H, s, 1-OCH_3_), 2.35 (1H, d, *J* = 12.5 Hz, H-4), 2.23 (1H, d, *J* = 14.0 Hz, H-4). ^13^C NMR (100 MHz, CDCl_3_) δ: 184.6 (C-7), 182.2 (C-12), 160.1 (C-8), 139.6 (C-6a), 137.3 (C-10), 133.6 (C-11a), 121.9 (C-9), 118.9 (C-11), 118.5 (C-5), 115.9 (C-12a), 113.8 (C-7a), 111.1 (C-4a), 83.9 (C-1), 77.6 (C-3)*, 77.4 (C-2)*, 59.1 (2-OCH_3_), 58.0 (1-OCH_3_), 56.7 (3-OCH_3_), 34.7 (C-12b), 31.3 (C-4). (*) interchangeable signals. HRMS (ES^+^): *m*/*z* calcd for C_20_H_21_NO_6_ [M + H]^+^: 372.1447; found 372.1450.

*(1R,2S,3R,12bR)-8,11-Dihydroxy-1,2,3-trimethoxy-1,2,3,4,6,12b-hexahydrobenzo[b]phenanthridine-7,12-dione* (**16c**). Purple solid (264.2 mg, 55%); mp 139.0 °C; IR (NaCl windows) cm^−1^ = 1608 and 1579 (ν C=O); 1498 (ν C=C). ^1^H NMR (400 MHz, CDCl_3_) δ: 13.23 (1H, s, 11-OH), 11.65 (1H, s, 8-OH), 7.15 (1H, d, *J* = 9.1 Hz, H-10)*, 7.05 (1H, d, *J* = 3.1 Hz, NH), 6.98 (1H, d, *J* = 9.1 Hz, H-9)*, 5.96 (1H, d, *J* = 3.4 Hz, H-5), 4.16 (1H, d, *J* = 10.2 Hz, H-12b), 3.73 (1H, bs, H-3), 3.66 (1H, bs, H-2), 3.49 (3H, s, 2-OCH_3_)^#^, 3.45 (1H, dd, *J* = 10.2, 2.0 Hz, H-1), 3.34 (3H, s, 1-OCH_3_) ^#^, 3.22 (3H, s, 3-OCH_3_) ^#^, 2.31 (1H, d, *J* = 13.8 Hz, H-4), 2.23 (1H, d, *J* = 13.8 Hz, H-4). (*,^#^) interchangeable signals. ^13^C NMR (100 MHz, CDCl_3_) δ: 186.36 (C-12), 182.14 (C-7), 157.0 (C-8), 155.5 (C-11), 140.8 (C-12a), 131.0 (C-9), 125.6 (C-10), 118.6 (C-5), 116.3 (C-4a), 111.3 (C-11a)*, 111.2(C-7a)*, 109.6 (C-6a), 83.2 (C-1), 77.5 (C-3)^#^, 76.5 (C-2)^#^, 58.7 (2-OCH_3_)^+^, 57.7 (1-OCH_3_) ^+^, 56.6 (3-OCH_3_) ^+^, 34.2 (C-12b), 31.1 (C-4). (*^,#,+^) interchangeable signals. HRMS (ES^+^): *m*/*z* calcd for C_20_H_21_NO_7_ [M + H]^+^: 388.1396; found 388.1396.

#### 3.1.5. General Procedure for the Synthesis of the Compounds **19**–**20**

Azadiene **13** (300 mg, 1.24 mmol) and appropriate bromonaphthoquinone (**18a**–**c**) (1.90 mmol) in anhydrous MeCN (30 mL) and NaHCO_3_ (42 mg, 0.50 mmol) were refluxed for 5 h. Removal of the solvent under reduced pressure resulted in a residue that was purified by column chromatography.

*(1S,2S)-1,2-Dimethoxy-1,2-dihydropyrido[3,2-b]phenanthridine-7,12-dione* (**19a**). Brown solid (115.9 mg, 29%); mp 78.8 °C; IR (NaCl windows) cm^−1^ = 1688 and 1665 (ν C=O); 1579 (ν C=N); 1279 (ν C-O). ^1^H NMR (400 MHz, CDCl_3_) δ: 8.88 (1H, s, H-5), 8.48 (1H, d, *J* = 8.2 Hz, H-11), 7.60 (1H, d, *J* = 8.2 Hz, H-10), 6.84 (1H, d, *J* = 9.6 Hz, H-4), 6.55 (1H, ddd, *J* = 9.6, 5.4, 0.8 Hz, H-3), 5.89 (1H, bs, H-1), 4.18 (1H, dd, *J* = 5.4, 1.8 Hz, H-2), 3.59 (3H, s, 1-OCH_3_), 3.44 (3H, s, 2-OCH_3_). ^13^C NMR (100 MHz, CDCl_3_) δ: 184.0 (C-12), 179.8 (C-7), 166.0 (C-9), 153.0 (C-5), 149.1 (C-6a), 147.5 (C-7a), 142.5 (C-12b), 135.7 (C-11), 132.4 (C-4a), 131.2 (C-3), 131.1 (C-11a), 128.3 (C-10), 128.0 (C-12a), 126.2 (C-4), 71.8 (C-2), 71.4 (C-1), 57.9 (1-OCH_3_), 56.9 (2-OCH_3_). HRMS (ES^+^): *m*/*z* calcd for C_18_H_14_N_2_O_4_ [M + H]^+^: 323.1032; found 323.1026.

*(1S,2S)-1,2-Dimethoxy-9-methyl-1,2-dihydropyrido[3,2-b]phenanthridine-7,12-dione* (**19b**). Brown solid (100.1 mg, 24%); mp 161.2 °C; IR (NaCl windows) cm^−1^ = 1693 and 1665 (ν C=O); 1584 (ν C=N); 1293 (ν C-O). ^1^H NMR (400 MHz, CDCl_3_) δ: 9.16 (1H, dd, *J* = 4.6, 1.4 Hz, H-9), 8.93 (1H, s, H-5), 8.65 (1H, dd, *J* = 7.9, 1.4 Hz, H-11), 7.80 (1H, dd, *J* = 7.9, 4.6 Hz, H-10), 6.88 (1H, d, *J* = 9.5 Hz, H-4), 6.59 (1H, dd, *J* = 9.5, 5.4 Hz, H-3), 5.92 (1H, bs, H-1), 4.22 (1H, dd, *J* = 5.4, 2.0 Hz, H-2), 3.63 (3H, s, 1-OCH_3_), 3.48 (3H, s, 2-OCH_3_), 2.81 (3H, s, 9-CH_3_). ^13^C NMR (100 MHz, CDCl_3_) δ 184.1 (C-12), 179.6 (C-7), 166.0 (C-9), 153.0 (C-5), 149.1 (C-6a), 147.5 (C-7a), 142.2 (C-12b), 135.7 (C-11), 132.1 (C-4a), 130.9 (C-3), 129.0 (C-11a), 128.4 (C-10), 127.8 (C-12a), 126.1 (C-4), 71.8 (C-2), 71.2 (C-1), 57.8 (1-OCH_3_), 56.8 (2-OCH_3_), 25.4 (9-CH_3_). HRMS (ES^+^): *m*/*z* calcd for C_19_H_16_N_2_O_4_ [M + H]^+^: 337.1178; found 337.1183.

*(1S,2S)-1,2-Dimethoxy-1,2-dihydrobenzo[b]phenanthridine-7,12-dione* (**19c**). Light yellow solid (119.5 mg, 30%); mp 117.5 °C; IR (NaCl windows) cm^−1^ = 1679 (ν C=O); 1588 (ν C=N); 1269 (ν C-O). ^1^H NMR (400 MHz, CDCl_3_) δ: 8.87 (1H, s, H-5), 8.38–8.34 (1H, m, H-8), 8.30–8.27 (1H, m, H-11), 7.85–7.81 (2H, m, H-9, H-10), 6.85 (1H, d, *J* = 9.6 Hz, H-4), 6.54 (1H, dd, *J* = 9.6, 5.4 Hz, H-3), 5.99 (1H, s, H-1), 4.19 (1H, dd, *J* = 5.4, 1.3 Hz, H-2), 3.61 (3H, s, 1-OCH_3_), 3.47 (3H, s, 2-OCH_3_). ^13^C NMR (100 MHz, CDCl_3_) δ: 184.8 (C-7), 181.1 (C-12), 152.7 (C-5), 149.3 (C-6a), 142.3 (C-12b), 134.5 (C-10), 134.4 (C-9), 134.1 (C-11a), 132.7 (C-7a), 132.1 (C-4a), 130.7 (C-3), 128.7 (C-12a), 127.5 (C-8), 127.4 (C-11), 126.3 (C-4), 72.0 (C-2), 71.2 (C-1), 57.8 (1-OCH_3_), 56.9 (2-OCH_3_). HRMS (ES^+^): *m*/*z* calcd for C_19_H_15_NO_4_ [M + H]^+^: 322.1079; found 322.1080.

*1-Methoxybenzo[b]phenanthridine-7,12-dione* (**20**). Yellow oil (64.6 mg, 18%); IR (NaCl windows) cm^−1^ = 1674 (ν C=O); 1593 (ν C=N); 1264 (ν C-O). ^1^H NMR (400 MHz, CDCl_3_) δ: 9.47 (1H, s, H-5), 8.34–8.32 (1H, m, H-8), 8.14–8.12 (1H, m, H-11), 7.83–7.75 (3H, m, H-10, H-9, H-3), 7.70–7.69 (1H, m, H-4), 7.33 (1H, d, *J* = 7.8 Hz, H-2), 4.04 (3H, s, 1-OCH_3_). ^13^C NMR (100 MHz, CDCl_3_) δ: 185.36 (C-7), 182.08 (C-12), 157.04 (C-5), 156.71 (C-1), 136.04 (C-6a), 134.23 (C-10), 133.26 (C-9), 132.02 (C-4b), 131.51 (C-3), 127.17 (C-8), 126.27 (C-11), 123.41 (C-12b), 120.31 (C-4), 113.22 (C-2), 56.33 (OCH_3_). HRMS (ES^+^): *m*/*z* calcd for C_18_H_11_NO_3_ [M + H]^+^: 290.0817; found 290.0810.

### 3.2. Biology

#### Cell Lines

The experimental cell lines were obtained from the American Type Culture Collection. HT-29 (colon cancer cell line), PC-3 (prostate cancer), MCF-7 (breast cancer), and CCD841 CoN (colon epithelial) were grown in DMEM-F12 containing 10% FCS, 100 U/mL penicillin, 100 µg/mL streptomycin, and 1 mM glutamine.

The in vitro cytotoxic activities of all the compounds were evaluated on cell lines previously mentioned by the sulforhodamine B assay, according to [42]. Briefly, cells were seeded at a plating density of 5 × 103 cells/well into 96 well plates. Cells were incubated at 37 °C for 24 h to allow cell attachment. The test compounds at indicated final concentrations were added to the culture medium and the cell cultures were continued for 72 h under the same conditions. Stock solutions of compounds were prepared in DMSO. Control cultures received 0.1% DMSO alone. At the end of the treatment, cells were fixed with trichloroacetic acid (50% *w*/*v*) at 4 °C and, subsequently, washed with water. Cells were stained with 0.1% sulforhodamine B in 1% acetic acid for 30 min. Posteriorly, the cells were washed acetic acid (1%) to remove unbound sulforhodamine B. Protein-bound stain was solubilized with 10 mM unbuffered Tris base). The optical density was determined using a fluorescence plate reader (540 nm). The obtained values are transformed to percentages of viable cells versus control treatment and the IC_50_ values calculated for each compound in the cell lines were studied using the SigmaPlot 12.0 software (Systat Software, San Jose, CA, USA). Values shown are the mean ± SD of the three independent experiments in triplicate.

## 4. Conclusions

The replacement of the ring A on the angucycline scaffold by (-)-shikimic acid led to new cytotoxic aza-analogous. Using the Diels-Alder reaction among naphthoquinones and an azadiene obtained from (-)-shikimic acid, nine angucyclines were achieved. The cytotoxic and selective effects on three-cancer cell lines by the synthesised compounds and five previously reported compounds depended on the chemical modifications of the angucycline system. Preliminary analysis confirmed that, mainly, the size of protected groups on ring A and the pattern of substitution on ring D are features that determine the cytotoxicity and selectivity of these compounds. Our results show that, depending on the cancer cell line, **7a**, **17**, **19a,** and **19c** are promising leads for the development of new antitumor drugs.

## Figures and Tables

**Table 1:** In vitro antitumor activity of angucyclinone *N*-analogues.

		IC_50_ Values (μM) ^a^			
Compound	Structure	PC-3	HT-29	MCF-7	CCD841-CoN
**7a**	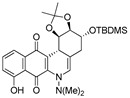	0.7 ± 0.1 ^b^ (3.3)	0.2 ± 0.05 (11.5)	16.0 ± 1.0 (0.1)	2.3 ± 0.3
**7b**	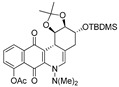	2.4 ± 0.9 (0.3)	2.1 ± 1.2 (0.4)	12.1 ± 4.0 (0.1)	0.8 ± 0.1
**8**	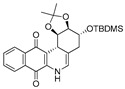	5.7 ± 1.3 (2.4)	7.8 ± 0.9 (1.8)	4.2 ± 4.2 (3.3)	13.8 ± 1.0
**9a**	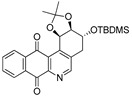	7.6 ± 1.2 (0.4)	3.7 ± 0.8 (0.8)	17.8 ± 3.0 (0.2)	2.8 ± 0.4
**9b**	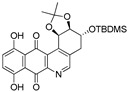	13.1 ± 2.0 (1.2)	15.0 ± 4.2 (1.1)	28.3 ± 4.0 (0.6)	15.6 ± 2.1
**15**	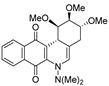	34.8 ± 0.3 (2.3)	29.2 ± 6.8 (2.8)	78.1 ± 6.9 (1.0)	81.6 ± 15.8
**16a**	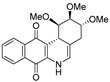	23.6 ± 3.7 (4.9)	42.9 ± 8.4 (2.7)	118.1 ± 10.8 (1.0)	115.7 ± 17.8
**16b**	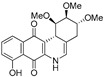	6.6 ± 1.1 (2.3)	5.3 ± 0.8 (2.9)	11.5 ± 3.1 (1.3)	15.5 ± 3.1
**16c**	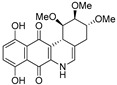	6.4 ± 0.8 (1.4)	4.0 ± 0.3 (2.2)	13.5 ± 0.9 (0.7)	8.8 ± 1.3
**17**	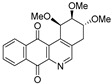	3.9 ± 0.6 (3.0)	3.3 ± 0.9 (3.6)	11.9 ± 1.5 (1.0)	11.8 ± 1.3
**19a**	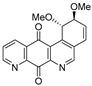	0.08 ± 0.01 (0.5)	0.10 ± 0.01 (0.4)	0.01± 0.005 (4.0)	0.04 ± 0.01
**19b**	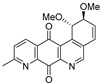	0.42 ± 0.04 (0.7)	0.39 ± 0.04 (0.8)	0.19 ± 0.04 (1.6)	0.30 ± 0.02
**19c**	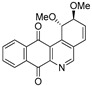	4.0 ± 0.4 (4.4)	21.0 ± 3.6 (0.8)	12.8 ± 1.4 (1.4)	17.4 ± 2.0
**20**	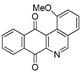	7.8 ± 1.0 (3.0)	3.7 ± 1.2 (6.4)	14.0 ± 1.7 (1.7)	23.6 ± 3.4

^a^ IC_50_ values were determined in three independent experiments for triplicate in the range of 1.25 to 100 µM. ^b^ In parentheses, the Selectivity index (SI) expressed as ratio of IC_50_ in CoN in cancer cells.

## Supplementary Materials

The following are available online. NMR spectra, IR spectra and HRMS of all compounds, and crystallographic data of **19c**.

## References

[1] Krohn K., Agocs A., Bäuerlein C. (2003). Total synthesis of angucyclines. XVII. First synthesis of antibiotic 100-1, a deoxydisaccharide angucycline antibiotic of the urdamycinone b-type. J. Carbohydr. Chem..

[2] Malmierca M.G., González-Montes L., Pérez-Victoria I., Sialer C., Braña A.F., García Salcedo R., Martín J., Reyes F., Méndez C., Olano C. (2018). Searching for glycosylated natural products in actinomycetes and identification of novel macrolactams and angucyclines. Front. Microbiol..

[3] Rohr J., Thiericke R. (1992). Angucycline group antibiotics. Nat. Prod. Rep..

[4] Xie Z.P., Liu B., Wang H.P., Yang S.X., Zhang H.Y., Wang Y.P., Ji N.Y., Qin S., Laatsch H. (2012). Kiamycin, a unique cytotoxic angucyclinone derivative from a marine *Streptomyces* sp.. Mar. Drugs.

[5] Guo Z.K., Liu S.B., Jiao R.H., Wang T., Tan R.X., Ge H.M. (2012). Angucyclines from an insect-derived *actinobacterium Amycolatopsis* sp. Hca1 and their cytotoxic activity. Bioorg. Med. Chem. Lett..

[6] Song Y., Liu G., Li J., Huang H., Zhang X., Zhang H., Ju J. (2015). Cytotoxic and antibacterial angucycline- and prodigiosin- analogues from the deep-sea derived *Streptomyces* sp. Scsio 11594. Mar Drugs.

[7] Ma M., Rateb M.E., Teng Q., Yang D., Rudolf J.D., Zhu X., Huang Y., Zhao L.-X., Jiang Y., Li X. (2015). Angucyclines and angucyclinones from *Streptomyces* sp. Cb01913 featuring c-ring cleavage and expansion. J. Nat. Prod..

[8] Kaliappan K.P., Ravikumar V. (2007). Angucyclinone antibiotics:  Total syntheses of YM-181741, (+)-ochromycinone, (+)-rubiginone B2, (−)-tetrangomycin, and MM-47755. J. Org. Chem..

[9] Bowie J.H., Johnson A.W. (1967). The structure of ochromycinone. Tetrahedron Lett..

[10] Oka M., Kamei H., Hamagishi Y., Tomita K., Miyaki T., Konishi M., Oki T. (1990). Chemical and biological properties of rubiginone, a complex of new antibiotics with vincristine-cytotoxicity potentiating activity. J. Antibiot..

[11] Rohr J., Zeeck A. (1987). Metabolic products of microorganisms. 240. Urdamycins, new angucycline antibiotics from *streptomyces fradiae*. II. Structural studies of urdamycins B to F. J. Antibiot..

[12] Puder C., Zeeck A., Beil W. (2000). New biologically active rubiginones from *Streptomyces* sp.. J. Antibiot..

[13] Park H.B., Lee J.K., Lee K.R., Kwon H.C. (2014). Angumycinones a and b, two new angucyclic quinones from *Streptomyces* sp. KMC004 isolated from acidic mine drainage. Tetrahedron Lett..

[14] Cone M.C., Hassan A.M., Gore M.P., Gould S.J., Borders D.B., Alluri M.R. (1994). Detection of phenanthroviridin aglycon in a UV mutant of *Streptomyces murayamaensis*. J. Org. Chem..

[15] Gore M.P., Gould S.J., Weller D.D. (1991). Total synthesis of phenanthroviridin aglycon: The first naturally-occurring benzo[*b*]phenanthridine. J. Org. Chem..

[16] Habbu P., Warad V., Shastri R., Madagundi S., Kulkarni V.H. (2016). Antimicrobial metabolites from marine microorganisms. Chin. J. Nat. Med..

[17] Zhang W., Liu Z., Li S., Lu Y., Chen Y., Zhang H., Zhang G., Zhu Y., Liu J., Zhang C. (2012). Fluostatins I-K from the south china sea-derived *Micromonospora rosaria* SCSIO N160. J. Nat. Prod..

[18] Doull J.L., Singh A.K., Hoare M., Ayer S.W. (1994). Conditions for the production of jadomycin-B by *Streptomyces-venezuelae* isp5230—Effects of heat-shock, ethanol treatment and phage infection. J. Ind. Microbiol..

[19] Ayer S.W., Mcinnes A.G., Thibault P., Walter J.A., Doull J.L., Parnell T., Vining L.C. (1991). Jadomycin, a novel 8*H*-benz[*b*]oxazolo[3,2-*f*]phenanthridine antibiotic from *Streptomyces-venezuelae* isp5230. Tetrahedron Lett..

[20] Hall S.R., Toulany J., Bennett L.G., Martinez-Farina C.F., Robertson A.W., Jakeman D.L., Goralski K.B. (2017). Jadomycins inhibit type II topoisomerases and promote DNA damage and apoptosis in multidrug-resistant triple-negative breast cancer cells. J. Pharmacol. Exp. Ther..

[21] Issa M.E., Hall S.R., Dupuis S.N., Graham C.L., Jakeman D.L., Goralski K.B. (2014). Jadomycins are cytotoxic to ABCB1-, ABCC1-, and ABCG2-overexpressing MCF7 breast cancer cells. Anti Cancer Drugs.

[22] Fan K., Zhang X., Liu H., Han H., Luo Y., Wang Q., Geng M., Yang K. (2012). Evaluation of the cytotoxic activity of new jadomycin derivatives reveals the potential to improve its selectivity against tumor cells. J. Antibiot..

[23] Kharel M.K., Pahari P., Shepherd M.D., Tibrewal N., Nybo S.E., Shaaban K.A., Rohr J. (2012). Angucyclines: Biosynthesis, mode-of-action, new natural products, and synthesis. Nat. Prod. Rep..

[24] Kesenheimer C., Kalogerakis A., Meißner A., Groth U. (2010). The cobalt way to angucyclinones: Asymmetric total synthesis of the antibiotics (+)-rubiginone B2, (−)-tetrangomycin, and (−)-8-*O*-methyltetrangomycin. Chem. Eur. J..

[25] Krohn K., Böker N., Flörke U., Freund C. (1997). Synthesis of angucyclines. 8. Biomimetic-type synthesis of rabelomycin, tetrangomycin, and related ring B aromatic angucyclinones. J. Org. Chem..

[26] Mal D., Dey S. (2006). Synthesis of chlorine-containing angucycline BE-23254 and its analogs. Tetrahedron.

[27] Landells J.S., Larsen D.S., Simpson J. (2003). Remote stereochemical control in asymmetric diels-alder reactions: Synthesis of the angucycline antibiotics, (−)-tetrangomycin and MM 47755. Tetrahedron Lett..

[28] Carreno M.C., Urbano A., Di Vitta C. (1999). Short and efficient enantioselective total synthesis of angucyclinone type antibiotics (+)-rubiginone B-2 and (+)-ochromycinone. Chem. Commun..

[29] Vu N.Q., Dujardin G., Collet S.C., Raiber E.-A., Guingant A.Y., Evain M. (2005). Synthesis of 5-aza-analogues of angucyclines: Manipulation of the 2-deoxy-*C*-glycoside subunit. Tetrahedron Lett..

[30] Sissouma D., Dequirez G., Collet S., Guingant A. (2011). Preparation of a chiral azadiene for the synthesis of 5-aza analogues of angucyclinones. Tetrahedron Lett..

[31] Valderrama J.A., Gonzalez M.F., Valderrama C. (1999). Studies on quinones. Part 32. Regioselective synthesis of benz[*b*]phenantridines related to phenantroviridone. Tetrahedron.

[32] Cuellar M.A., Quinones N., Vera V., Salas C.O., Estevez J.C., Estevez R.J. (2015). Preliminary studies on the synthesis of (−)-shikimic acid based 1,2,3,4-tetrahydrobenzo[*b*]phenanthridine-7,12-diones. Synlett.

[33] Valderrama J.A., Vasquez D. (2008). Design and synthesis of angucyclinone ab-pyrido[2,3-*d*] pyrimidine analogues. Tetrahedron Lett..

[34] Valderrama J.A., Colonelli P., Vasquez D., Gonzalez M.F., Rodriguez J.A., Theoduloz C. (2008). Studies on quinones. Part 44: Novel angucyclinone *N*-heterocyclic analogues endowed with antitumoral activity. Bioorg. Med. Chem..

[35] Jeso V., Iqbal S., Hernandez P., Cameron M.D., Park H., LoGrasso P.V., Micalizio G.C. (2013). Synthesis of benzoquinone ansamycin-inspired macrocyclic lactams from shikimic acid. Angew. Chem. Int. Ed. Engl..

[36] Vazquez K., Espinosa-Bustos C., Soto-Delgado J., Tapia R.A., Varela J., Birriel E., Segura R., Pizarro J., Cerecetto H., Gonzalez M. (2015). New aryloxy-quinone derivatives as potential antichagasic agents: Synthesis, trypanosomicidal activity, electrochemical properties, pharmacophore elucidation and 3D-QSAR analysis. RSC Adv..

[37] Cuellar M.A., Alegria L.K., Prieto Y.A., Cortes M.J., Tapia R.A., Preite M.D. (2002). Hetero-diels-alder reaction of halogenated quinones with a polygodial-derived azadiene. Tetrahedron Lett..

[38] Nebois P., do Nascimento S.C., Boitard M., Bartoli M.H., Fillion H. (1994). Synthesis and in vitro cytotoxic activity of aza- and diazaanthraquinone derivatives. Pharmazie.

[39] Poumaroux A., Bouaziz Z., Fillion H., Domard M., Giraud J., Petavy A.F. (1999). Regiospecific synthesis of pyrido[3,4-*b*]- and pyrido[4,3-*b*]carbazole-5,11-dione derivatives. Evaluation of their in vitro antifungal or antiprotozoological activities. Chem. Pharm. Bull..

[40] Salas C.O., Faundez M., Morello A., Maya J.D., Tapia R.A. (2011). Natural and synthetic naphthoquinones active against *Trypanosoma cruzi*: An initial step towards new drugs for chagas disease. Curr. Med. Chem..

[41] Sieveking I., Thomas P., Estevez J.C., Quinones N., Cuellar M.A., Villena J., Espinosa-Bustos C., Fierro A., Tapia R.A., Maya J.D. (2014). 2-phenylaminonaphthoquinones and related compounds: Synthesis, trypanocidal and cytotoxic activities. Bioorg. Med. Chem..

[42] Skehan P., Storeng R., Scudiero D., Monks A., McMahon J., Vistica D., Warren J.T., Bokesch H., Kenney S., Boyd M.R. (1990). New colorimetric cytotoxicity assay for anticancer-drug screening. J. Natl. Cancer Inst..

