# Supplementary material for: (-)-Shikimic Acid as a Chiral Building Block for the Synthesis of New Cytotoxic 6-Aza-Analogues of Angucyclinones
Source: Molecules. 2018 Jun 12;23(6):1422. doi: 10.3390/molecules23061422 (PMC6099682; doi:10.3390/molecules23061422)
Supplement: Supplementary file 1 [file molecules-23-01422-s001.pdf]

molecules

MDPI

Supporting information

(-)-Shikimic acid as a chiral building block for the synthesis of new cytotoxic 6-aza-analogues of angucyclinones

Natalia Quiñones Sobarzo [1], Santiago Hernández [1,2], Luis Espinoza Catalán [2], Joan Villena García [3], Ivan Brito [4], Alan R. Cabrera [5], Cristian O. Salas [5*], Mauricio A. Cuellar [1*]

[1] Centro de Investigación Farmacopea Chilena, Escuela de Química y Farmacia, Facultad de Farmacia, Universidad de Valparaíso, Av. Gran Bretaña N° 1093, Valparaíso 2340000, Chile E-Mail: natalia.quinones@uv.cl.; santiago.hernandez@postgrado.uv.cl; mauricio.cuellar@uv.cl.

[2] Departamento de Química, Universidad Técnica Federico Santa María, Av. España N° 1680, Valparaíso 2340000, Chile; E-Mail: luis.espinozac@usm.cl.

[3] Centro de Investigaciones Biomédicas, Escuela de Medicina, Universidad de Valparaíso, Av. Hontaneda N° 2664, Valparaíso 2340000, Chile; E-Mail: juan.villena@uv.cl.

[4] Departamento de Química, Facultad de Ciencias Básicas, Universidad de Antofagasta, Av. Angamos 601, Antofagasta, Chile; E-Mail: ivan.brito@uantof.cl.

[5] Departamento de Química Orgánica, Facultad de Química, Pontificia Universidad Católica de Chile, Vicuña Mackenna N° 4860, Santiago 6094411, Chile; E-Mail: cosalas@uc.cl.

* Correspondence: cosalas@uc.cl -mauricio.cuellar@uv.cl; Tel.: +56-32-2508147

Supplementary Material

S.1 Nuclear magnetic resonance spectra

S.2 High resolution mass spectrometry

S.3 Infrared spectroscopy

S.1 Nuclear magnetic resonance spectra

Compound 11

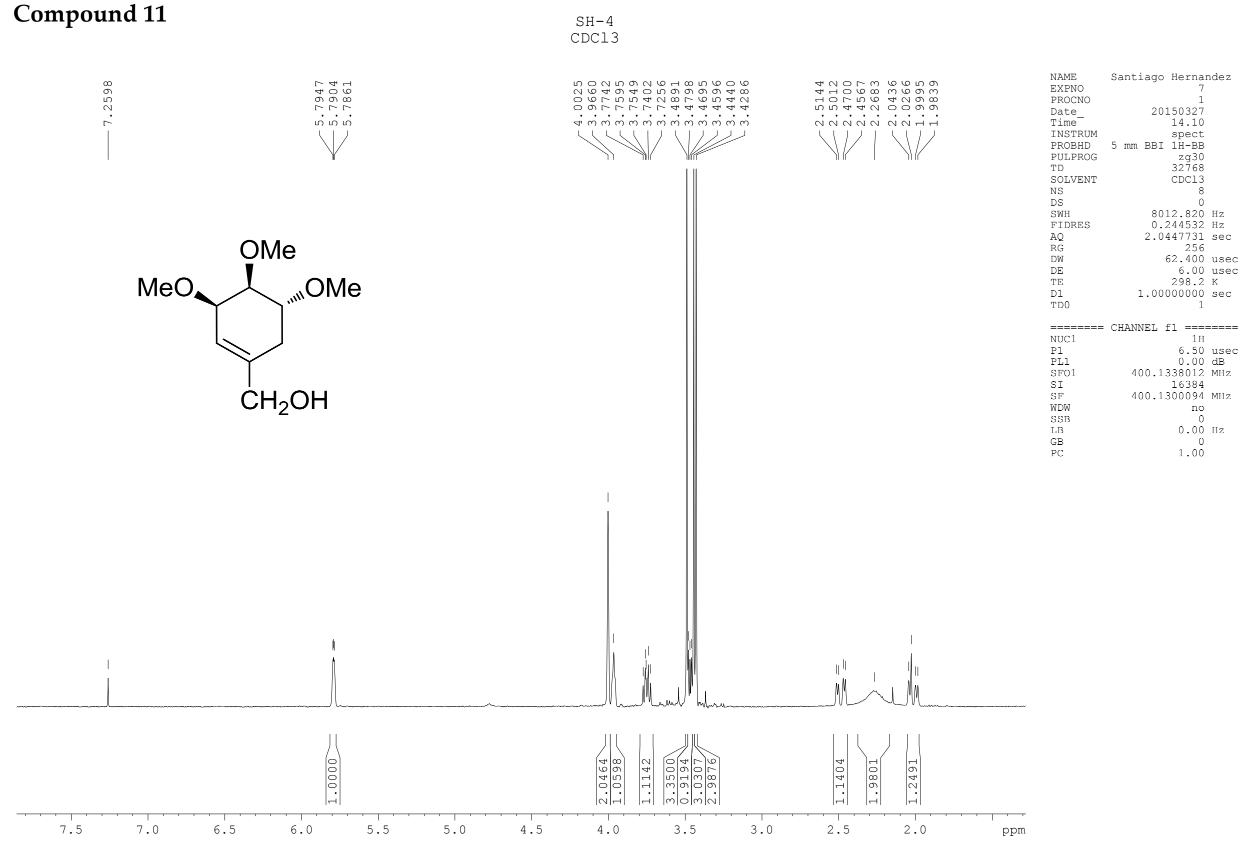

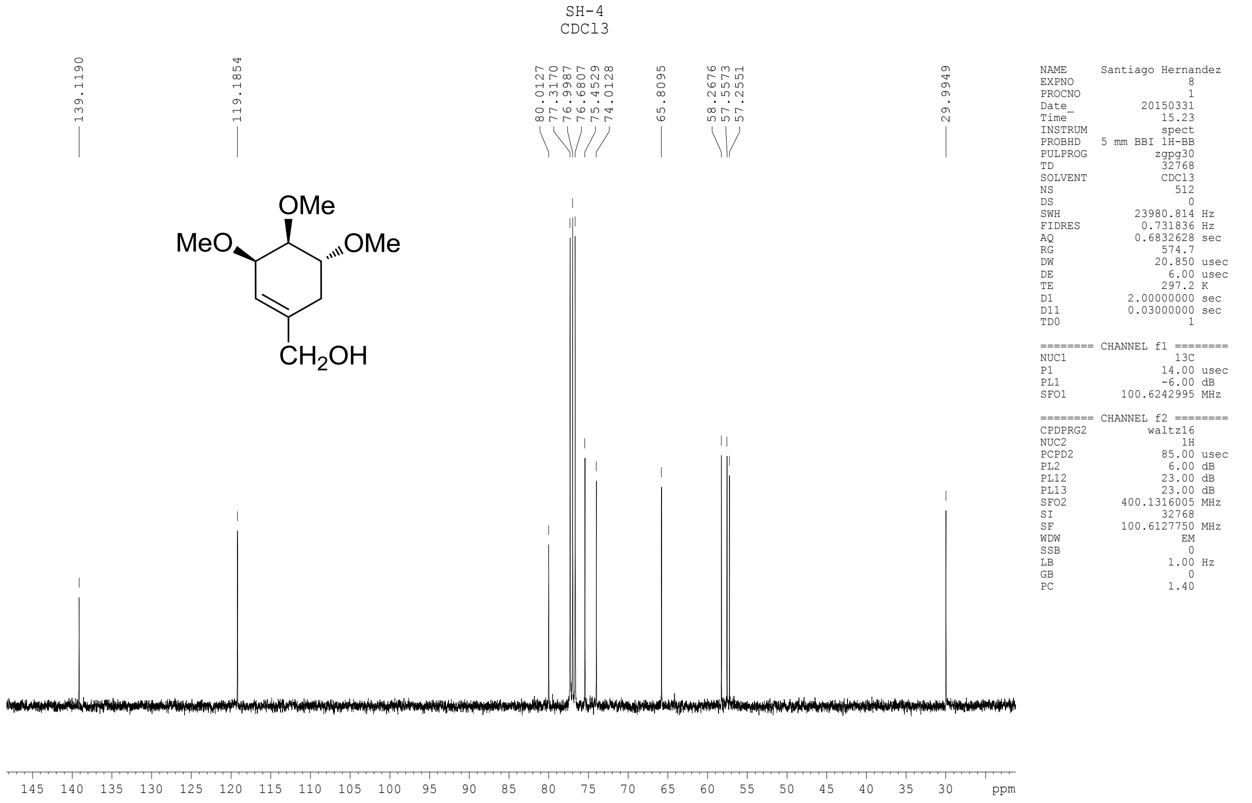

Compound 12

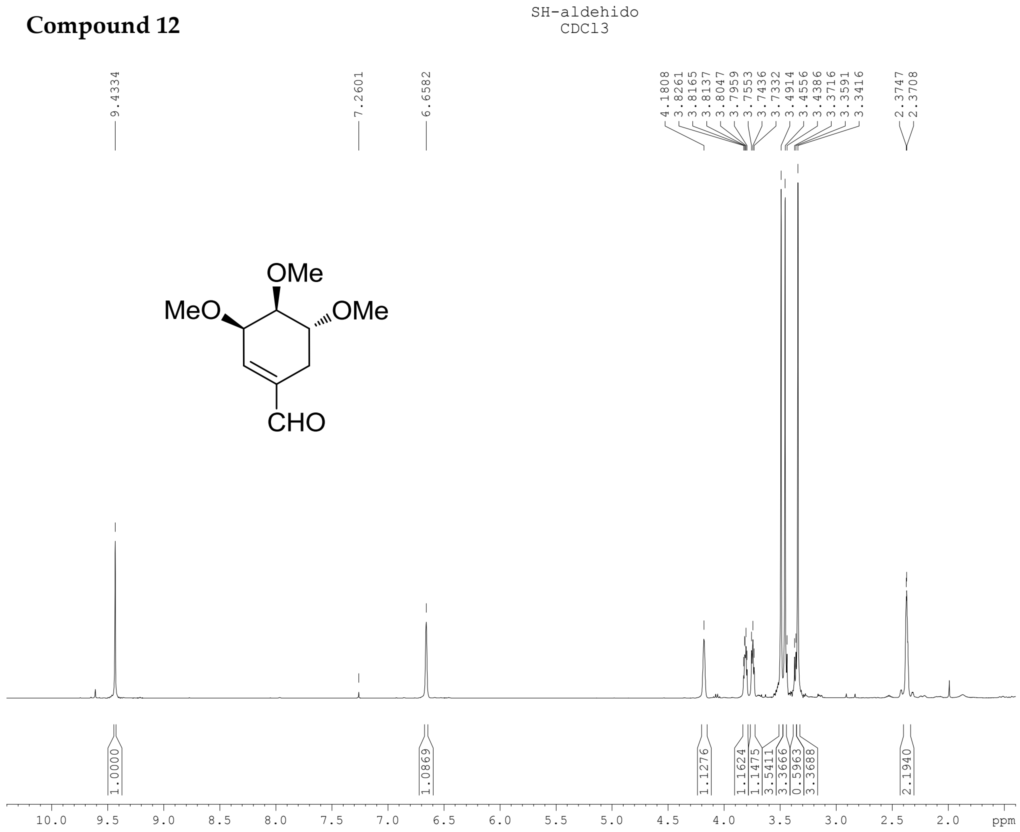


```
NAME      Santiago Hernandez
EXPNO                    14
PROCNO                    1
Date_              20150508
Time                  10.11
INSTRUM               spect
PROBHD   5 mm BBI 1H-BB
PULPROG                zg30
TD                    32768
SOLVENT               CDCl3
NS                        8
DS                        0
SWH               8012.820 Hz
FIDRES            0.244532 Hz
AQ               2.0447731 sec
RG                     71.8
DW                   62.400 usec
DE                     6.00 usec
TE                    296.2 K
D1              1.00000000 sec
TD0                       1

======== CHANNEL f1 ========
NUC1                     1H
P1                     6.50 usec
PL1                    0.00 dB
SFO1          400.1338012 MHz
SI                    16384
SF            400.1300089 MHz
WDW                      no
SSB                       0
LB                     0.00 Hz
GB                        0
PC                     1.00
```

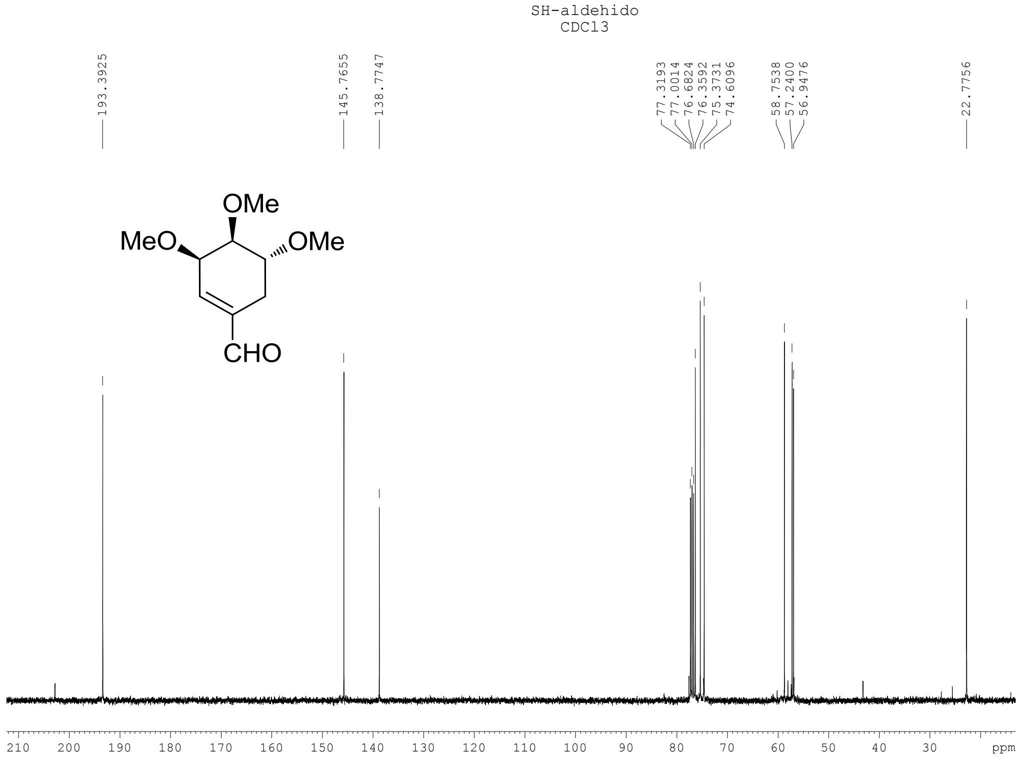


```
NAME      Santiago Hernandez
EXPNO                    23
PROCNO                    1
Date_              20150512
Time                  15.43
INSTRUM               spect
PROBHD   5 mm BBI 1H-BB
PULPROG              zgpg30
TD                    32768
SOLVENT               CDCl3
NS                      512
DS                        0
SWH              23980.814 Hz
FIDRES            0.731836 Hz
AQ               0.6832628 sec
RG                    574.7
DW                   20.850 usec
DE                     6.00 usec
TE                    296.2 K
D1              2.00000000 sec
D11             0.03000000 sec
TD0                       1

======== CHANNEL f1 ========
NUC1                    13C
P1                    14.00 usec
PL1                   -6.00 dB
SFO1          100.6242995 MHz

======== CHANNEL f2 ========
CPDPRG2             waltz16
NUC2                     1H
PCPD2                 85.00 usec
PL2                    6.00 dB
PL12                  23.00 dB
PL13                  23.00 dB
SFO2          400.1316005 MHz
SI                    32768
SF            100.6127804 MHz
WDW                      EM
SSB                       0
LB                     1.00 Hz
GB                        0
PC                     1.40
```

Compound 13

SH-6
CDCl3

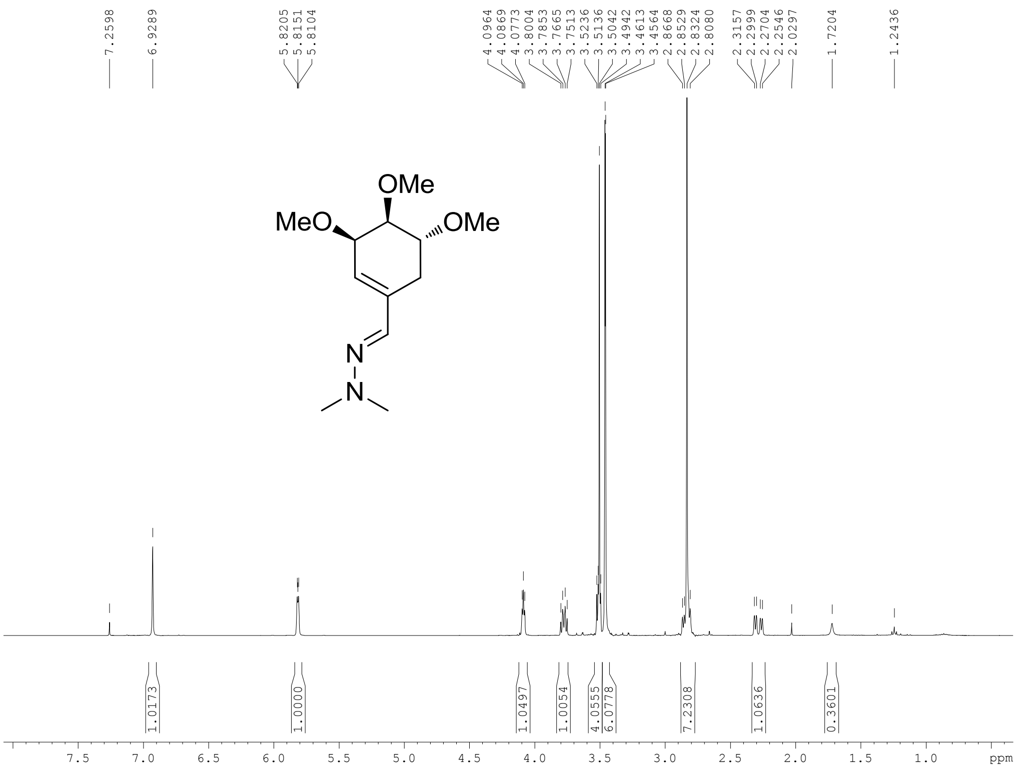

NAME	Santiago Hernandez
EXPNO	19
PROCNO	1
Date_	20150508
Time	10.41
INSTRUM	spect
PROBHD	5 mm BBI 1H-BB
PULPROG	zg30
TD	32768
SOLVENT	CDCl3
NS	8
DS	0
SWH	8012.820 Hz
FIDRES	0.244532 Hz
AQ	2.0447731 sec
RG	181
DW	62.400 usec
DE	6.00 usec
TE	296.2 K
D1	1.00000000 sec
TD0	1
========	CHANNEL f1 ========
NUC1	1H
P1	6.50 usec
PL1	0.00 dB
SFO1	400.1338012 MHz
SI	16384
SF	400.1300094 MHz
WDW	no
SSB	0
LB	0.00 Hz
GB	0
PC	1.00

SH-6
CDCl3

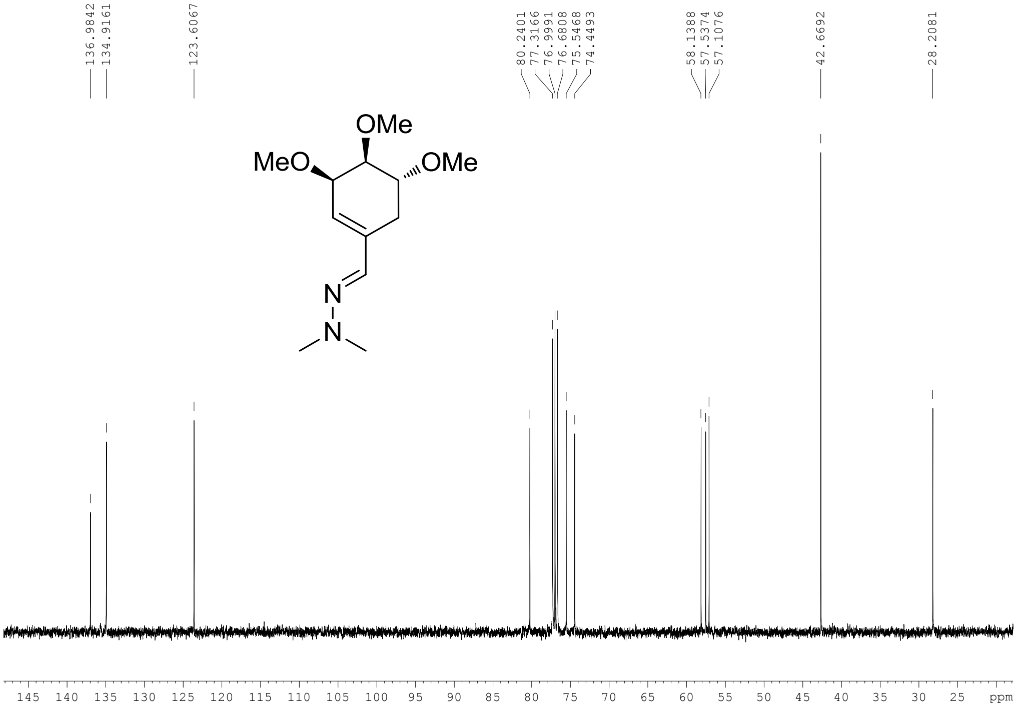

NAME	Santiago Hernandez
EXPNO	20
PROCNO	1
Date_	20150512
Time	14.52
INSTRUM	spect
PROBHD	5 mm BBI 1H-BB
PULPROG	zgpg30
TD	32768
SOLVENT	CDCl3
NS	512
DS	0
SWH	23980.814 Hz
FIDRES	0.731836 Hz
AQ	0.6832628 sec
RG	574.7
DW	20.850 usec
DE	6.00 usec
TE	296.2 K
D1	2.00000000 sec
D11	0.03000000 sec
TD0	1
========	CHANNEL f1 ========
NUC1	13C
P1	14.00 usec
PL1	-6.00 dB
SFO1	100.6242995 MHz
========	CHANNEL f2 ========
CPDPRG2	waltz16
NUC2	1H
PCPD2	85.00 usec
PL2	6.00 dB
PL12	23.00 dB
PL13	23.00 dB
SFO2	400.1316005 MHz
SI	32768
SF	100.6127733 MHz
WDW	EM
SSB	0
LB	1.00 Hz
GB	0
PC	1.40

Compound 15

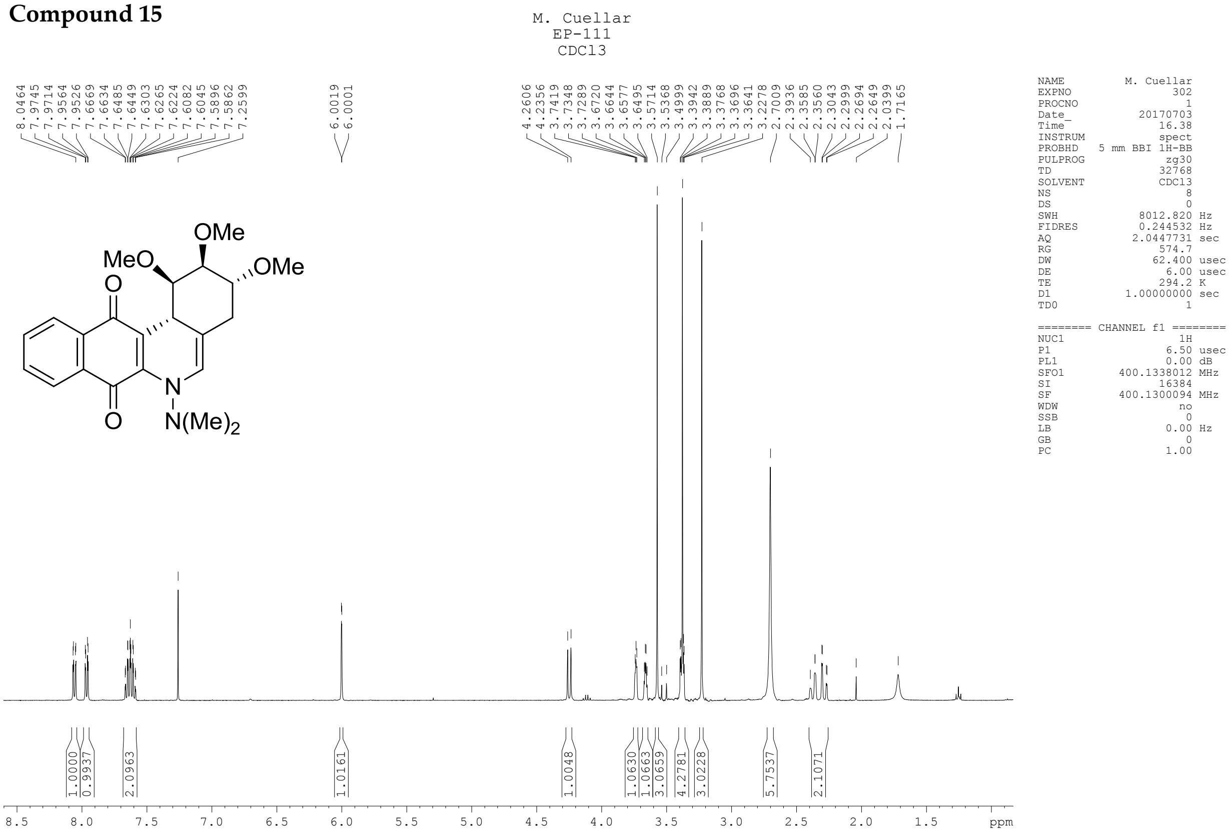
M. Cuellar
EP-111
CDCl3
8.0464 7.9745 7.9714 7.9564 7.9526 7.6669 7.6634 7.6485 7.6449 7.6303 7.6265 7.6224 7.6082 7.6045 7.5896 7.5862 7.2599
6.0019 6.0001
4.2606 4.2356 3.7419 3.7348 3.7289 3.6720 3.6644 3.6577 3.6495 3.5714 3.5368 3.4999 3.3942 3.3889 3.3768 3.3696 3.3641 3.2278 2.7009 2.3936 2.3585 2.3560 2.3043 2.2999 2.2694 2.2649 2.0399 1.7165
OMe
MeO
OMe
O
N
O
N(Me)2
NAME M. Cuellar
EXPNO 302
PROCNO 1
Date_ 20170703
Time 16.38
INSTRUM spect
PROBHD 5 mm BBI 1H-BB
PULPROG zg30
TD 32768
SOLVENT CDCl3
NS 8
DS 0
SWH 8012.820 Hz
FIDRES 0.244532 Hz
AQ 2.0447731 sec
RG 574.7
DW 62.400 usec
DE 6.00 usec
TE 294.2 K
D1 1.00000000 sec
TD0 1
======== CHANNEL f1 ========
NUC1 1H
P1 6.50 usec
PL1 0.00 dB
SFO1 400.1338012 MHz
SI 16384
SF 400.1300094 MHz
WDW no
SSB 0
LB 0.00 Hz
GB 0
PC 1.00
1.0000 0.9937 2.0963 1.0161 1.0048 1.0630 1.0663 3.0659 4.2781 3.0228 5.7537 2.1071
8.5 8.0 7.5 7.0 6.5 6.0 5.5 5.0 4.5 4.0 3.5 3.0 2.5 2.0 1.5 ppm

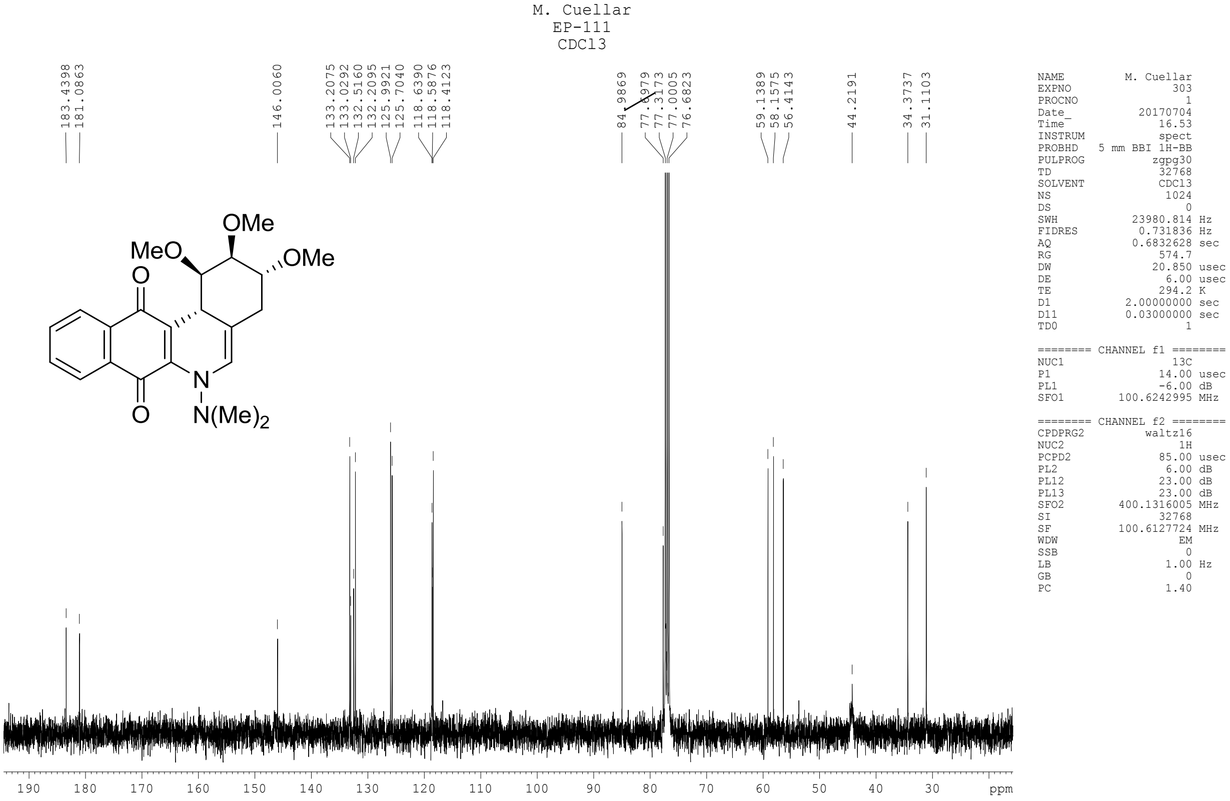
M. Cuellar
EP-111
CDCl3
183.4398 181.0863
146.0060
133.2075 133.0292 132.5160 132.2095 125.9921 125.7040 118.6390 118.5876 118.4123
84.9869 77.6979 77.3173 77.0005 76.6823
59.1389 58.1575 56.4143
44.2191
34.3737 31.1103
OMe
MeO
OMe
O
N
O
N(Me)2
NAME M. Cuellar
EXPNO 303
PROCNO 1
Date_ 20170704
Time 16.53
INSTRUM spect
PROBHD 5 mm BBI 1H-BB
PULPROG zgpg30
TD 32768
SOLVENT CDCl3
NS 1024
DS 0
SWH 23980.814 Hz
FIDRES 0.731836 Hz
AQ 0.6832628 sec
RG 574.7
DW 20.850 usec
DE 6.00 usec
TE 294.2 K
D1 2.00000000 sec
D11 0.03000000 sec
TD0 1
======== CHANNEL f1 ========
NUC1 13C
P1 14.00 usec
PL1 -6.00 dB
SFO1 100.6242995 MHz
======== CHANNEL f2 ========
CPDPRG2 waltz16
NUC2 1H
PCPD2 85.00 usec
PL2 6.00 dB
PL12 23.00 dB
PL13 23.00 dB
SFO2 400.1316005 MHz
SI 32768
SF 100.6127724 MHz
WDW EM
SSB 0
LB 1.00 Hz
GB 0
PC 1.40
190 180 170 160 150 140 130 120 110 100 90 80 70 60 50 40 30 ppm

Compound 16a

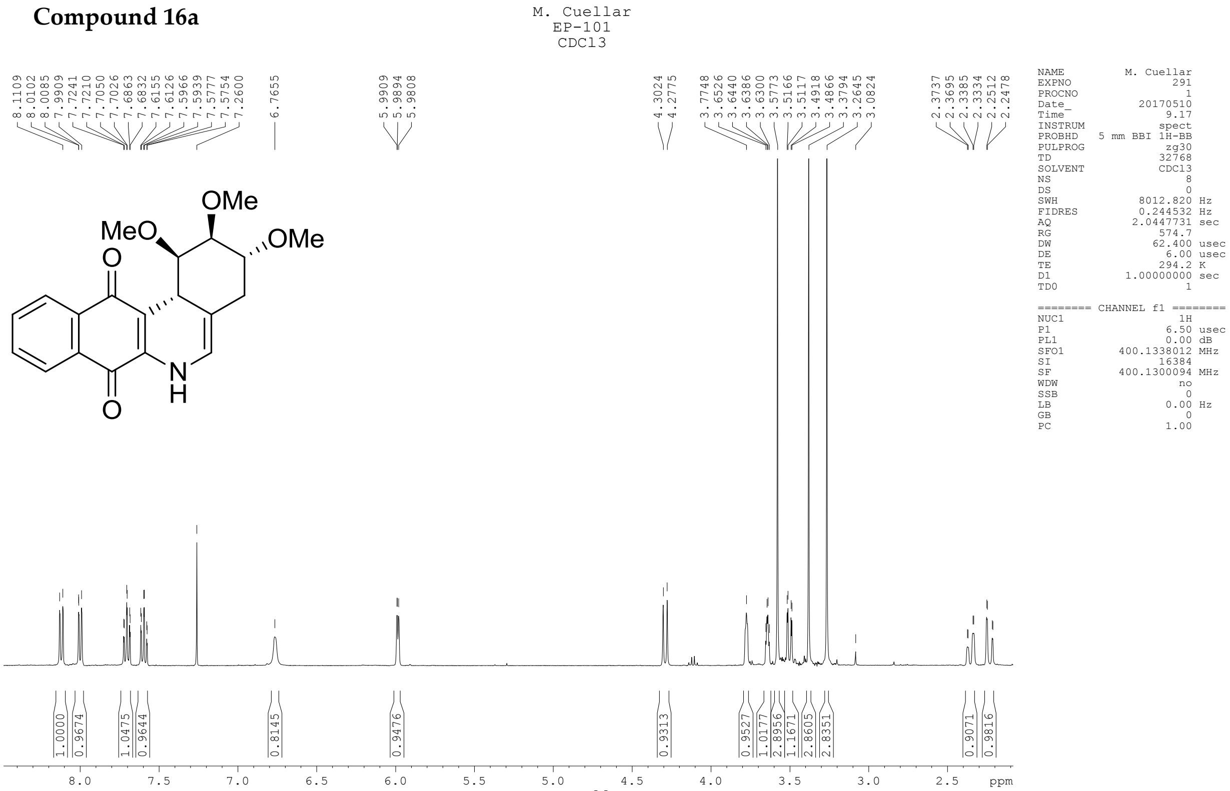

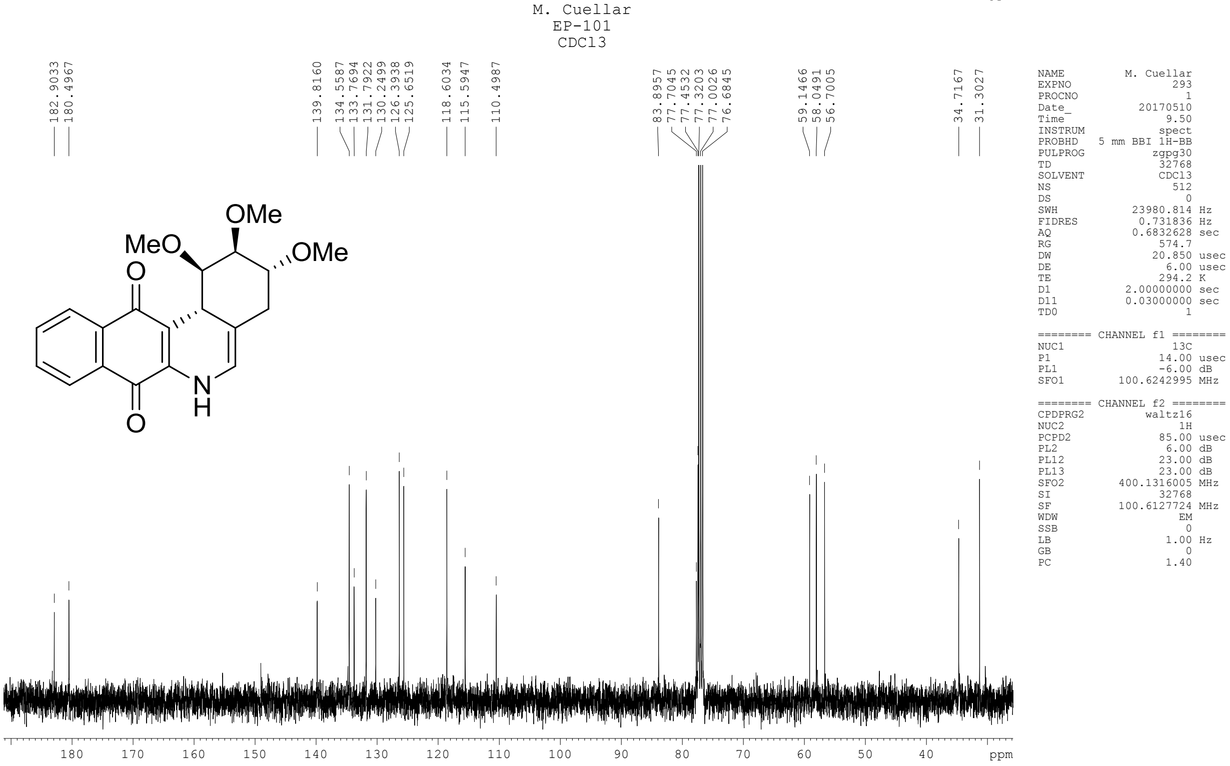

Juglona 2
CDCl3

Compound 16b

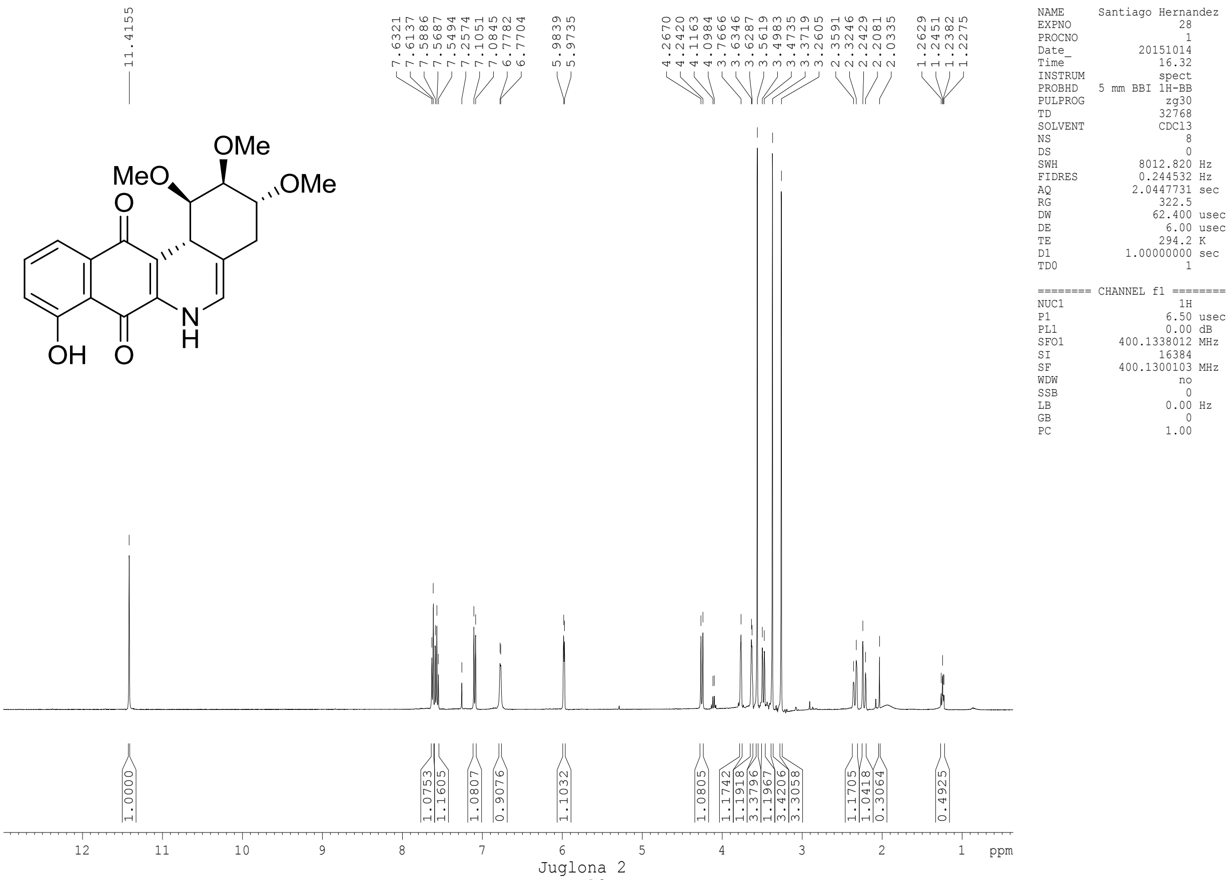

Juglona 2
CDCl3

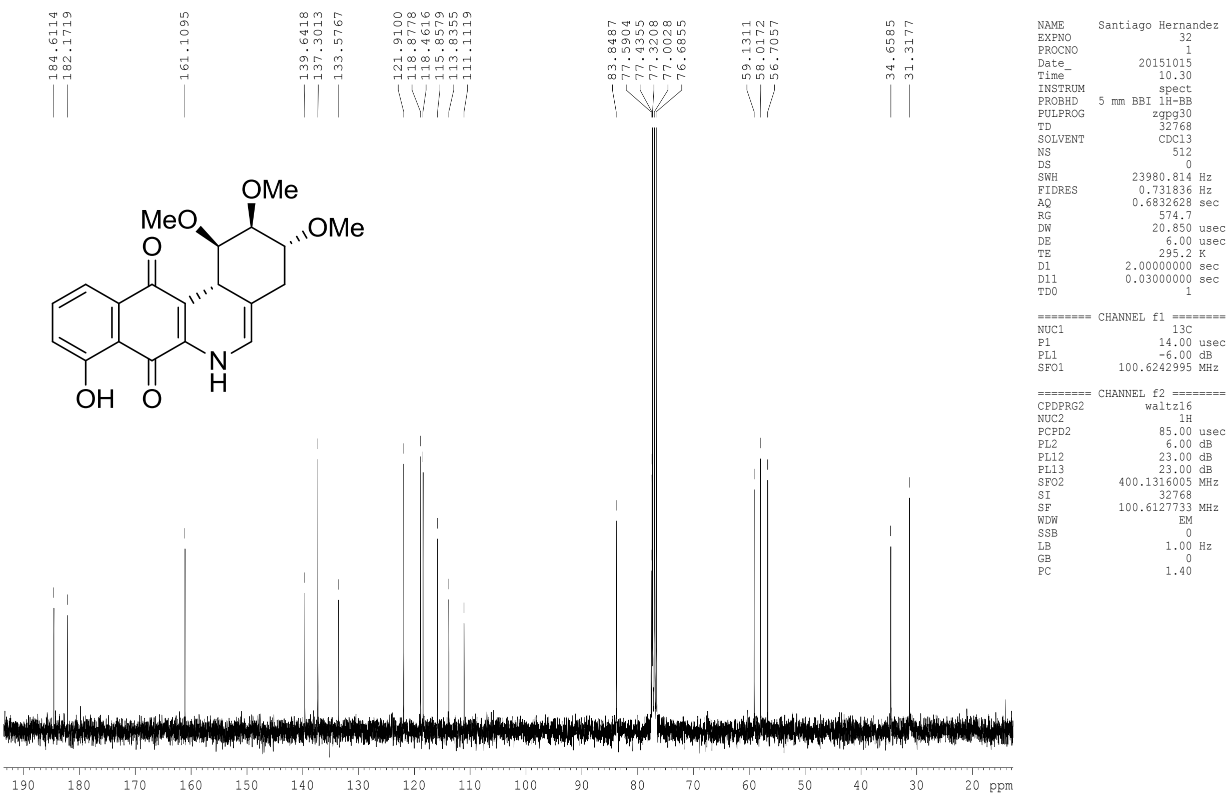

Compound 16c

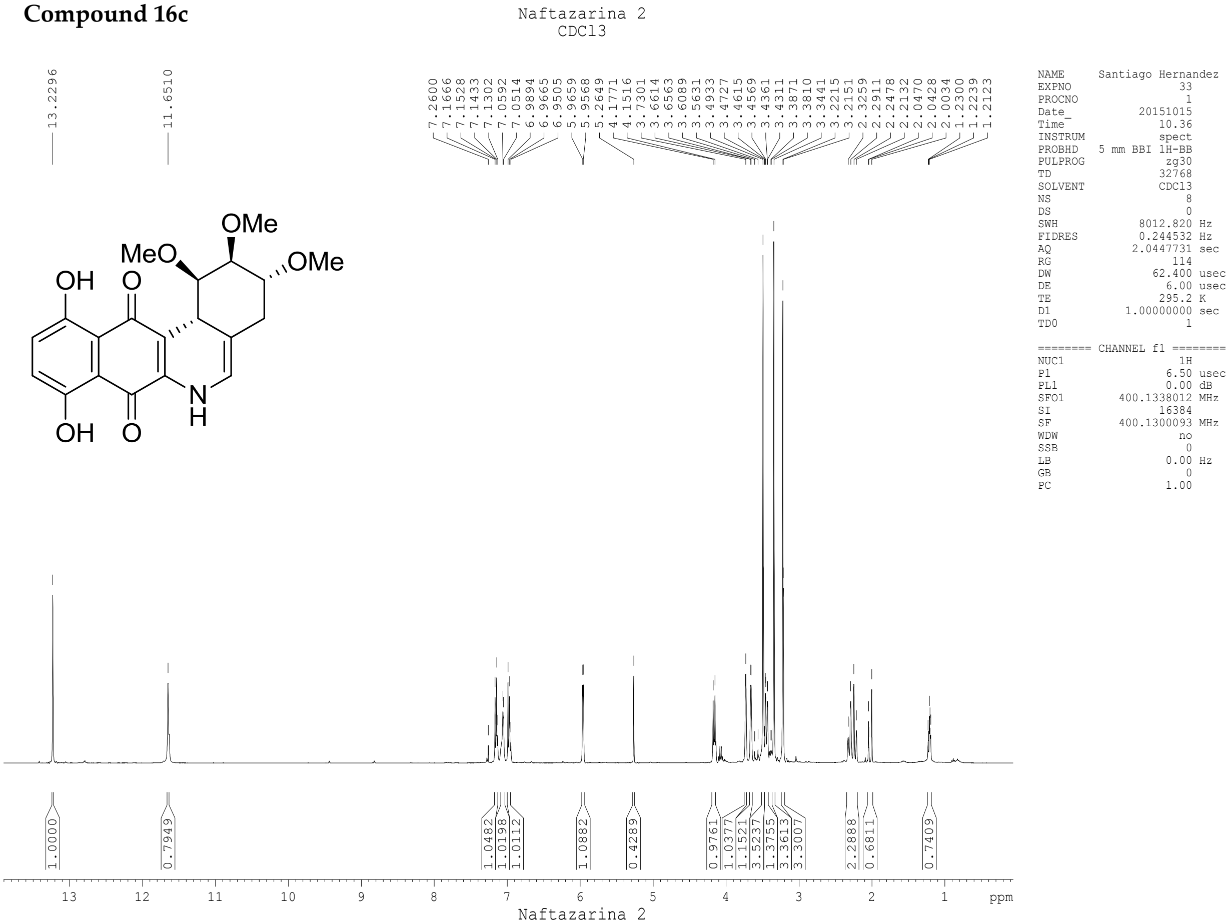

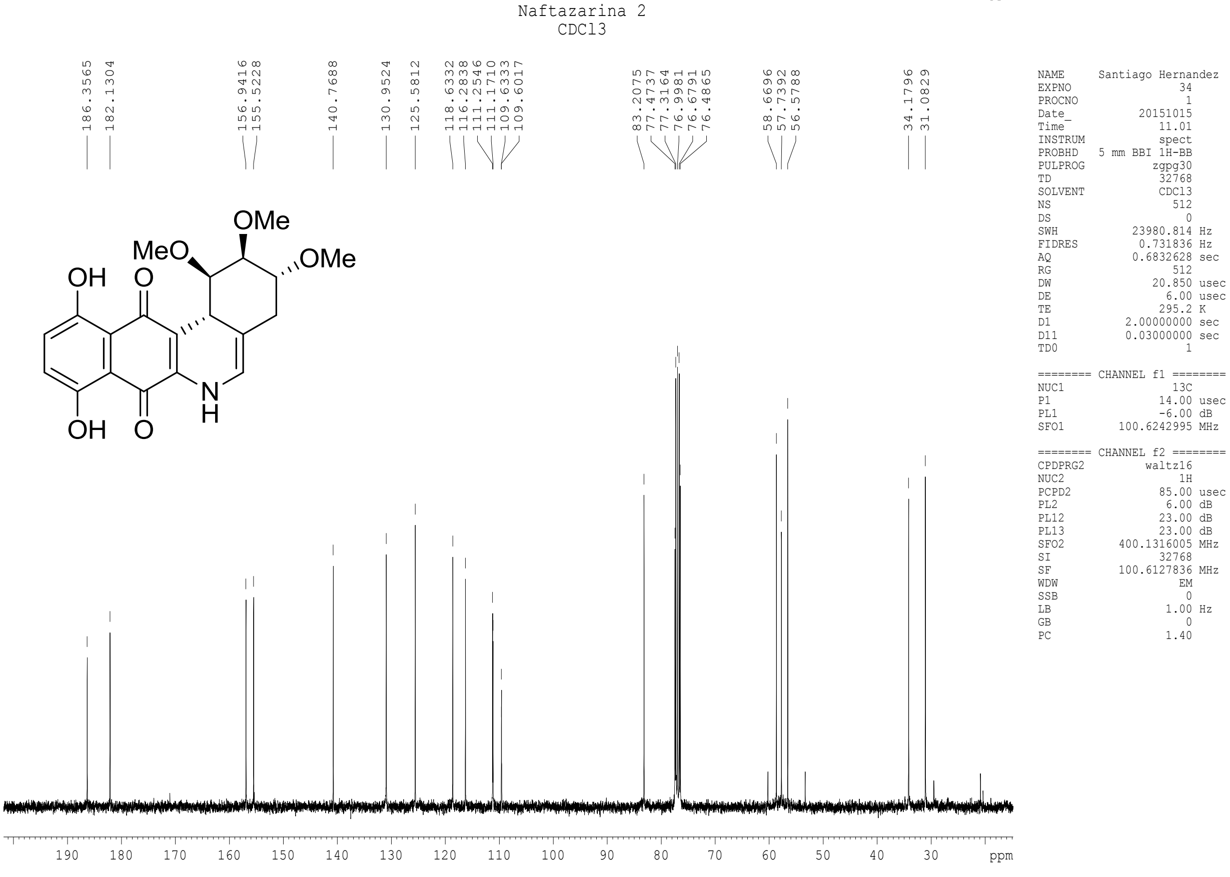

Compound 17

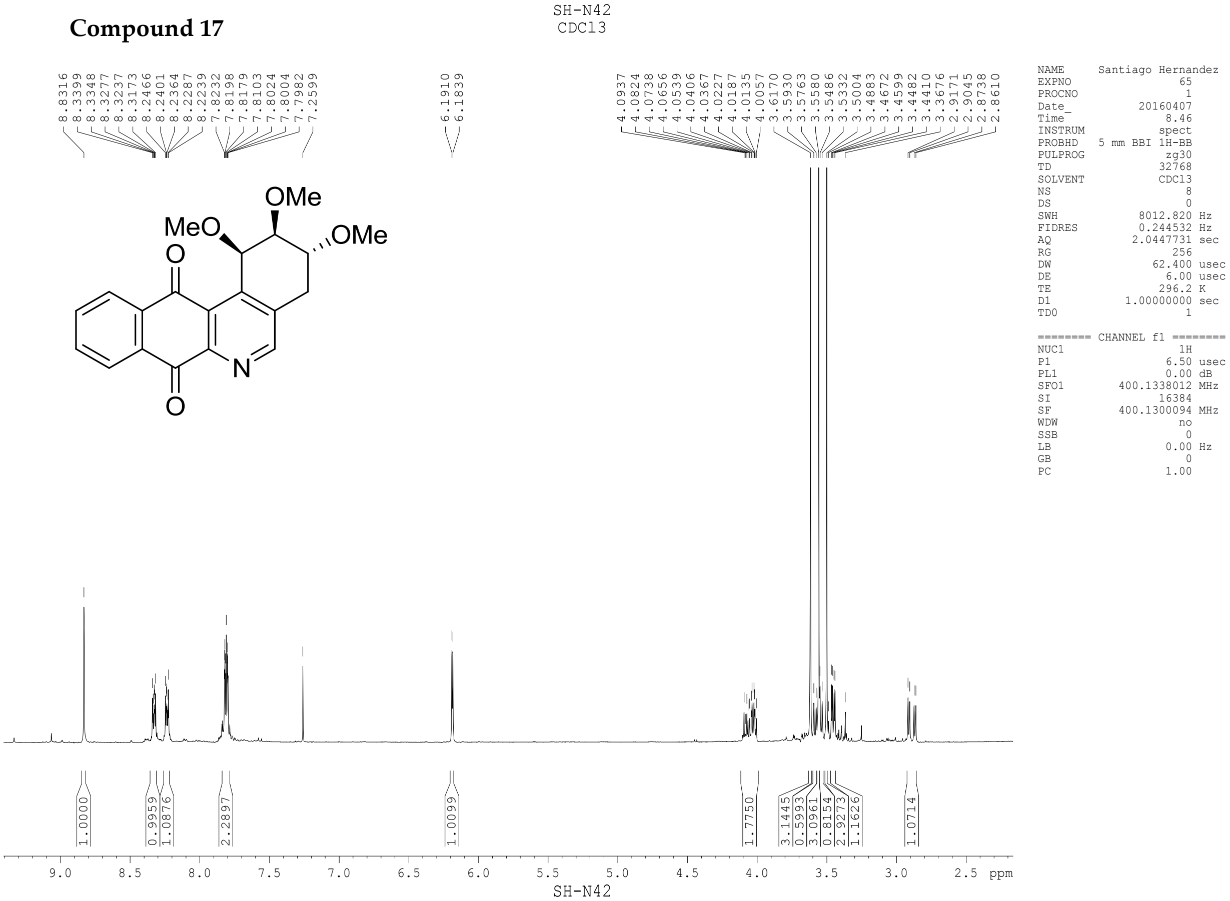
SH-N42
CDCl3
8.8316
8.3399
8.3348
8.3277
8.3237
8.3173
8.2466
8.2401
8.2364
8.2287
8.2239
7.8232
7.8198
7.8179
7.8103
7.8024
7.8004
7.7982
7.2599
6.1910
6.1839
4.0937
4.0824
4.0738
4.0656
4.0539
4.0406
4.0367
4.0227
4.0187
4.0135
4.0057
3.6170
3.5930
3.5763
3.5580
3.5486
3.5332
3.5004
3.4883
3.4672
3.4599
3.4482
3.4410
3.3676
2.9171
2.9045
2.8738
2.8610
OMe
MeO
OMe
O
N
O
1.0000
0.9959
1.0876
2.2897
1.0099
1.7750
3.1445
0.5993
3.0961
0.8154
2.9273
1.1626
1.0714
9.0 8.5 8.0 7.5 7.0 6.5 6.0 5.5 5.0 4.5 4.0 3.5 3.0 2.5 ppm
NAME Santiago Hernandez
EXPNO 65
PROCNO 1
Date_ 20160407
Time 8.46
INSTRUM spect
PROBHD 5 mm BBI 1H-BB
PULPROG zg30
TD 32768
SOLVENT CDCl3
NS 8
DS 0
SWH 8012.820 Hz
FIDRES 0.244532 Hz
AQ 2.0447731 sec
RG 256
DW 62.400 usec
DE 6.00 usec
TE 296.2 K
D1 1.00000000 sec
TD0 1
======== CHANNEL f1 ========
NUC1 1H
P1 6.50 usec
PL1 0.00 dB
SFO1 400.1338012 MHz
SI 16384
SF 400.1300094 MHz
WDW no
SSB 0
LB 0.00 Hz
GB 0
PC 1.00

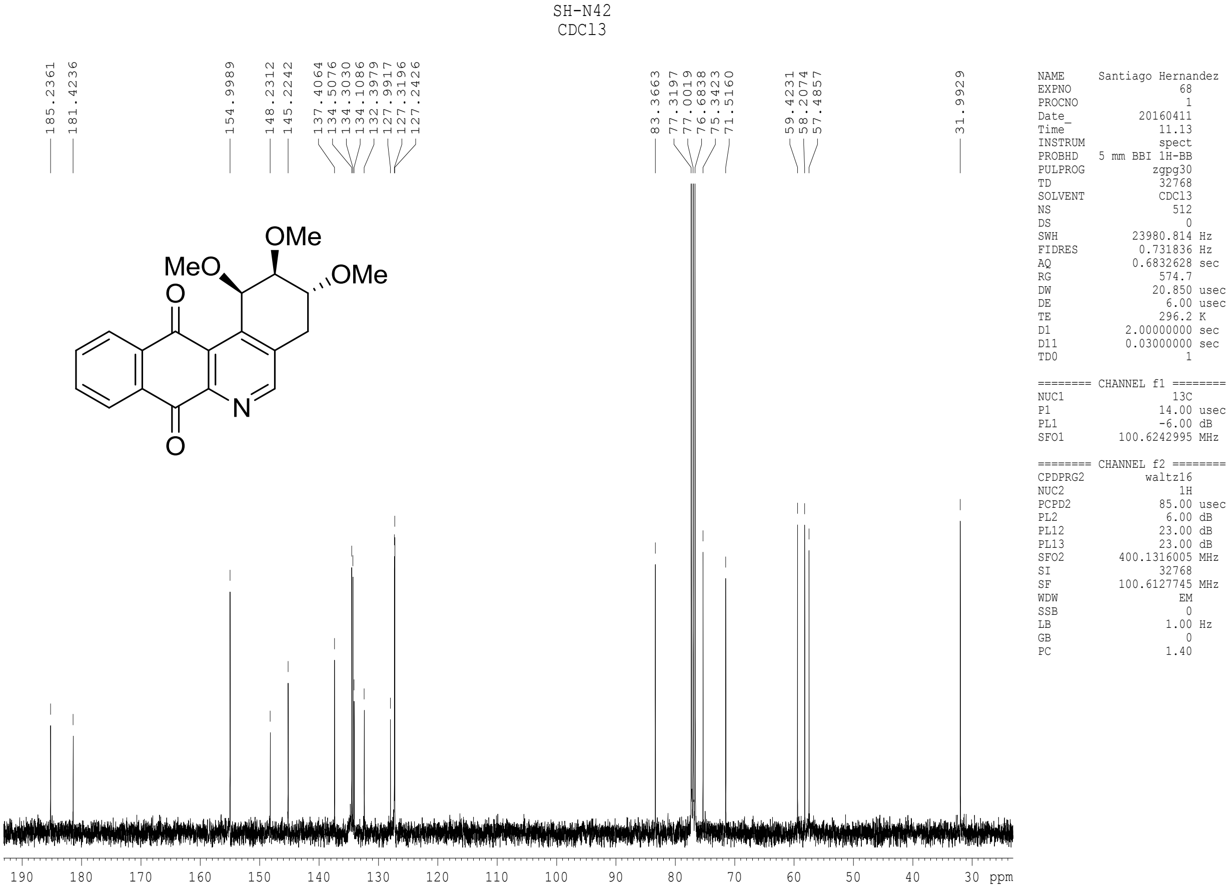
SH-N42
CDCl3
185.2361
181.4236
154.9989
148.2312
145.2242
137.4064
134.5076
134.3030
134.1086
132.3979
127.9917
127.3196
127.2426
83.3663
77.3197
77.0019
76.6838
75.3423
71.5160
59.4231
58.2074
57.4857
31.9929
OMe
MeO
OMe
O
N
O
190 180 170 160 150 140 130 120 110 100 90 80 70 60 50 40 30 ppm
NAME Santiago Hernandez
EXPNO 68
PROCNO 1
Date_ 20160411
Time 11.13
INSTRUM spect
PROBHD 5 mm BBI 1H-BB
PULPROG zgpg30
TD 32768
SOLVENT CDCl3
NS 512
DS 0
SWH 23980.814 Hz
FIDRES 0.731836 Hz
AQ 0.6832628 sec
RG 574.7
DW 20.850 usec
DE 6.00 usec
TE 296.2 K
D1 2.00000000 sec
D11 0.03000000 sec
TD0 1
======== CHANNEL f1 ========
NUC1 13C
P1 14.00 usec
PL1 -6.00 dB
SFO1 100.6242995 MHz
======== CHANNEL f2 ========
CPDPRG2 waltz16
NUC2 1H
PCPD2 85.00 usec
PL2 6.00 dB
PL12 23.00 dB
PL13 23.00 dB
SFO2 400.1316005 MHz
SI 32768
SF 100.6127745 MHz
WDW EM
SSB 0
LB 1.00 Hz
GB 0
PC 1.40

LE-MC2

Compound 19a

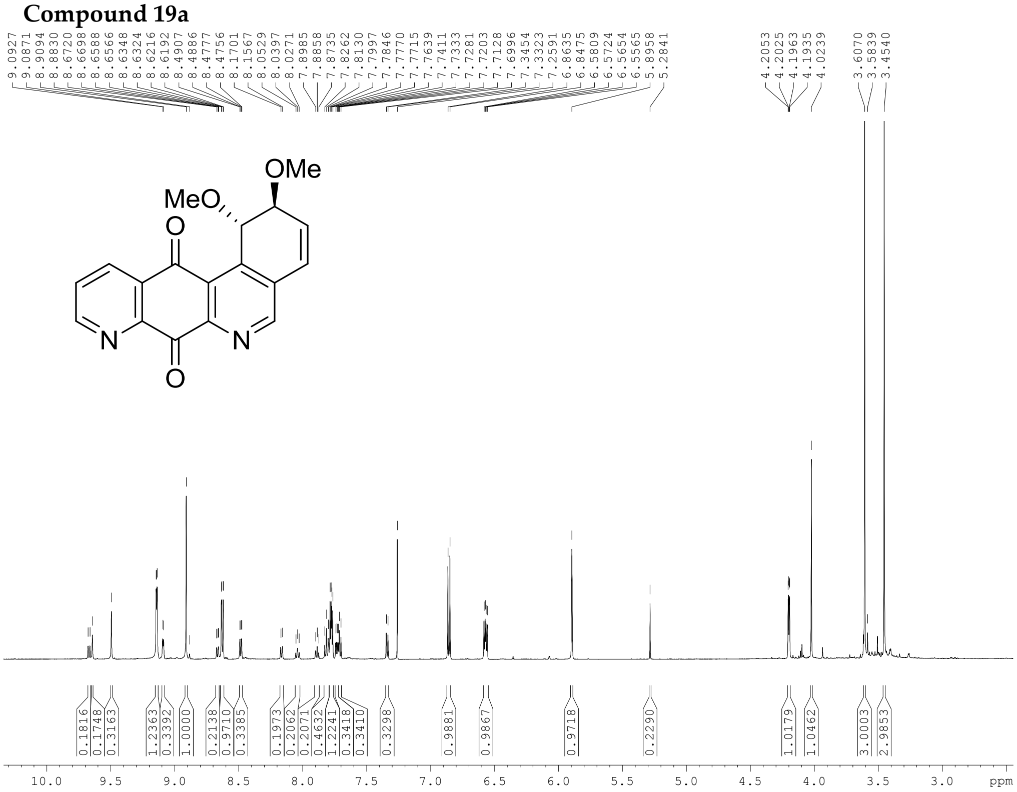

NAME le-mc2
EXPNO 1
PROCNO 1
Date_ 20171122
Time 7.19
INSTRUM spect
PROBHD 5 mm CPTCI 1H-
PULPROG zg30
TD 32768
SOLVENT CDCl3
NS 8
DS 2
SWH 12019.230 Hz
FIDRES 0.366798 Hz
AQ 1.3631988 sec
RG 12.7
DW 41.600 usec
DE 20.00 usec
TE 300.0 K
D1 1.00000000 sec
TD0 1

======== CHANNEL f1 ========
SFO1 600.2330011 MHz
NUC1 1H
P1 6.60 usec
SI 65536
SF 600.2300150 MHz
WDW no
SSB 0
LB 0.00 Hz
GB 0
PC 1.00

LE-MC2

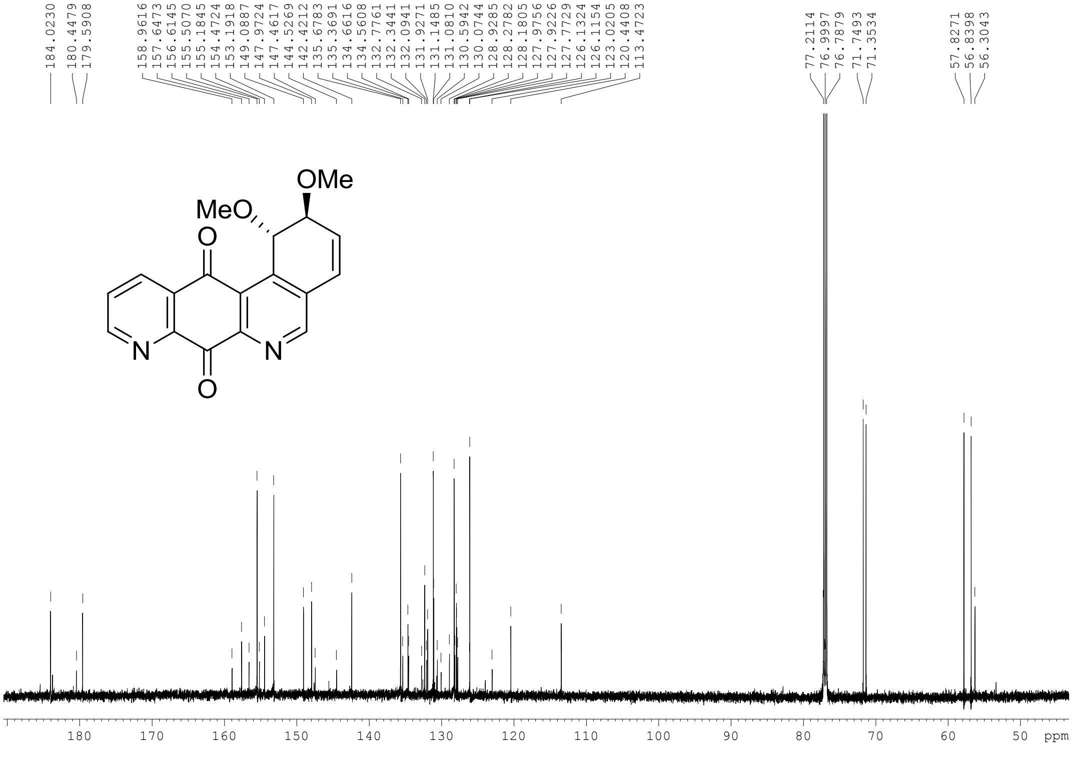

NAME le-mc2
EXPNO 2
PROCNO 1
Date_ 20171122
Time 7.31
INSTRUM spect
PROBHD 5 mm CPTCI 1H-
PULPROG jagzgdc30
TD 32768
SOLVENT CDCl3
NS 268
DS 0
SWH 34722.223 Hz
FIDRES 1.059638 Hz
AQ 0.4719092 sec
RG 1290
DW 14.400 usec
DE 15.00 usec
TE 300.0 K
D1 1.50000000 sec
D11 0.03000000 sec

======== CHANNEL f1 ========
SFO1 150.9445561 MHz
NUC1 13C
P1 9.50 usec
SI 131072
SF 150.9279599 MHz
WDW EM
SSB 0
LB 0.30 Hz
GB 0
PC 1.40

Compound 19b

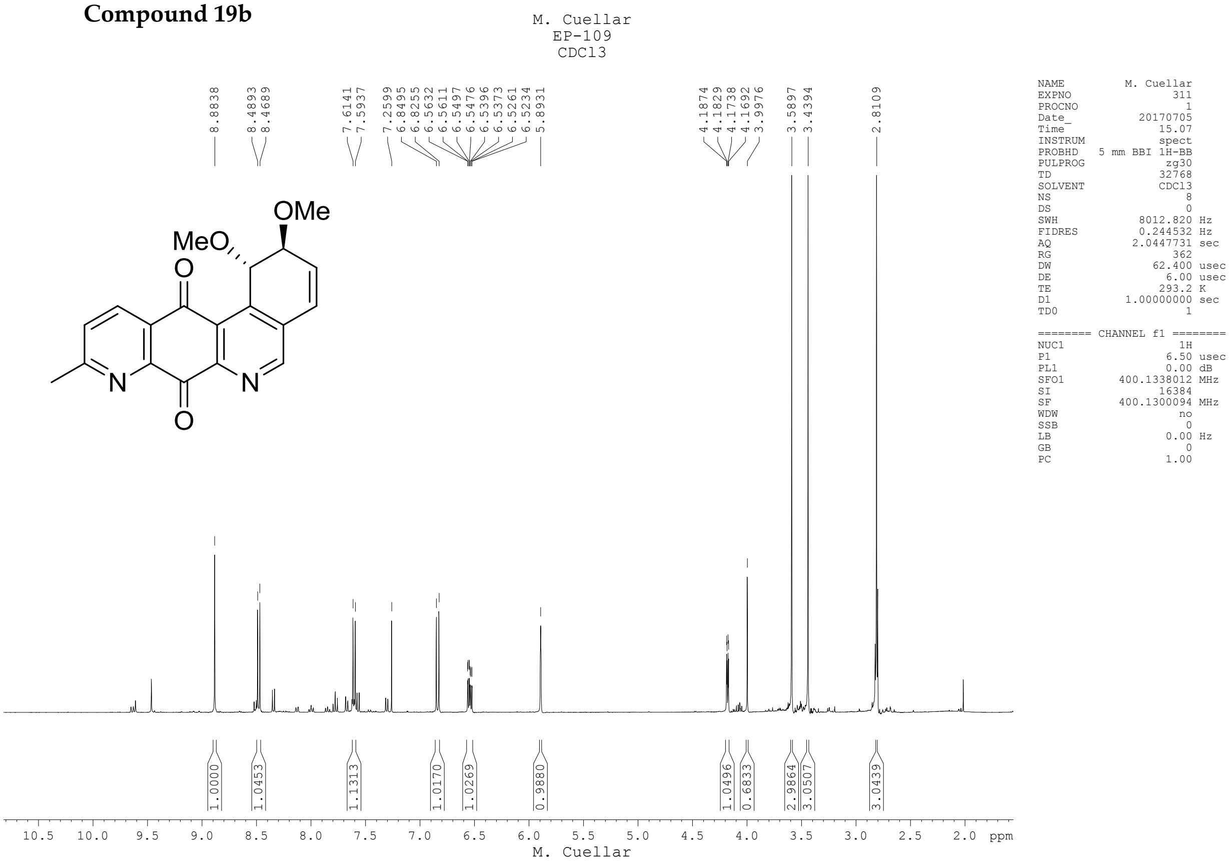

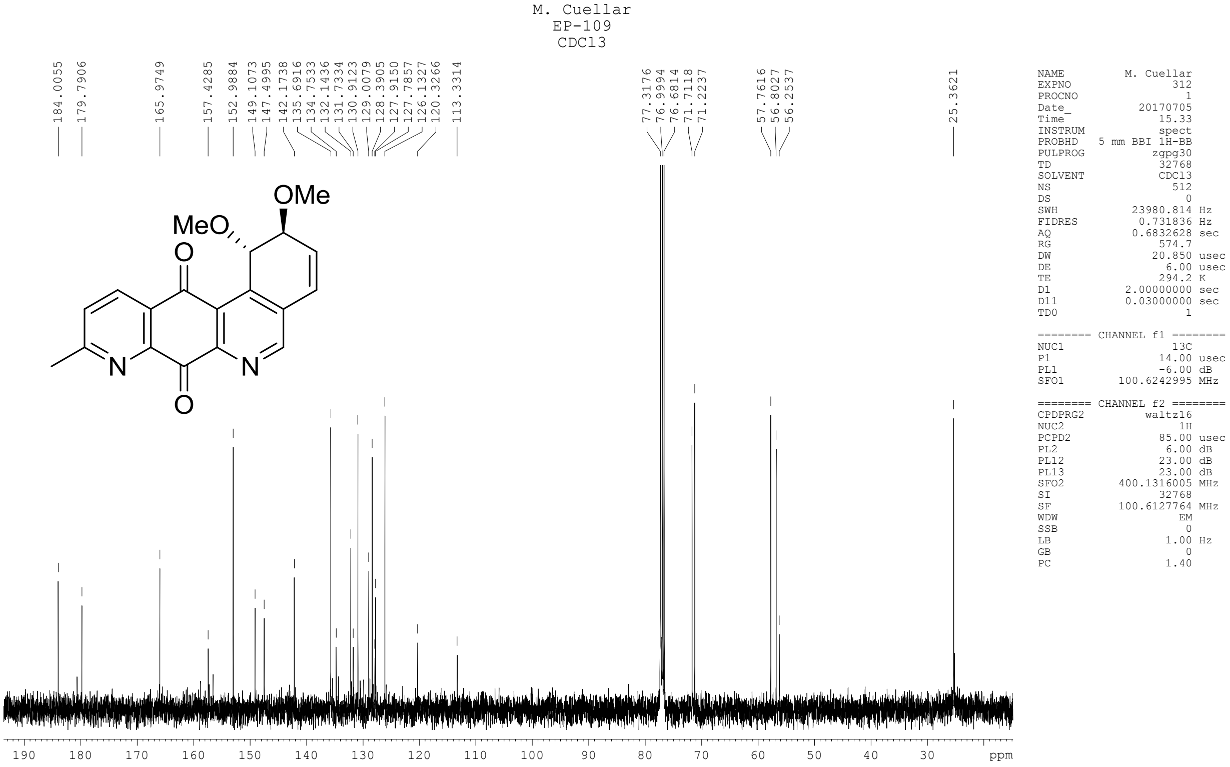

Compound 19c

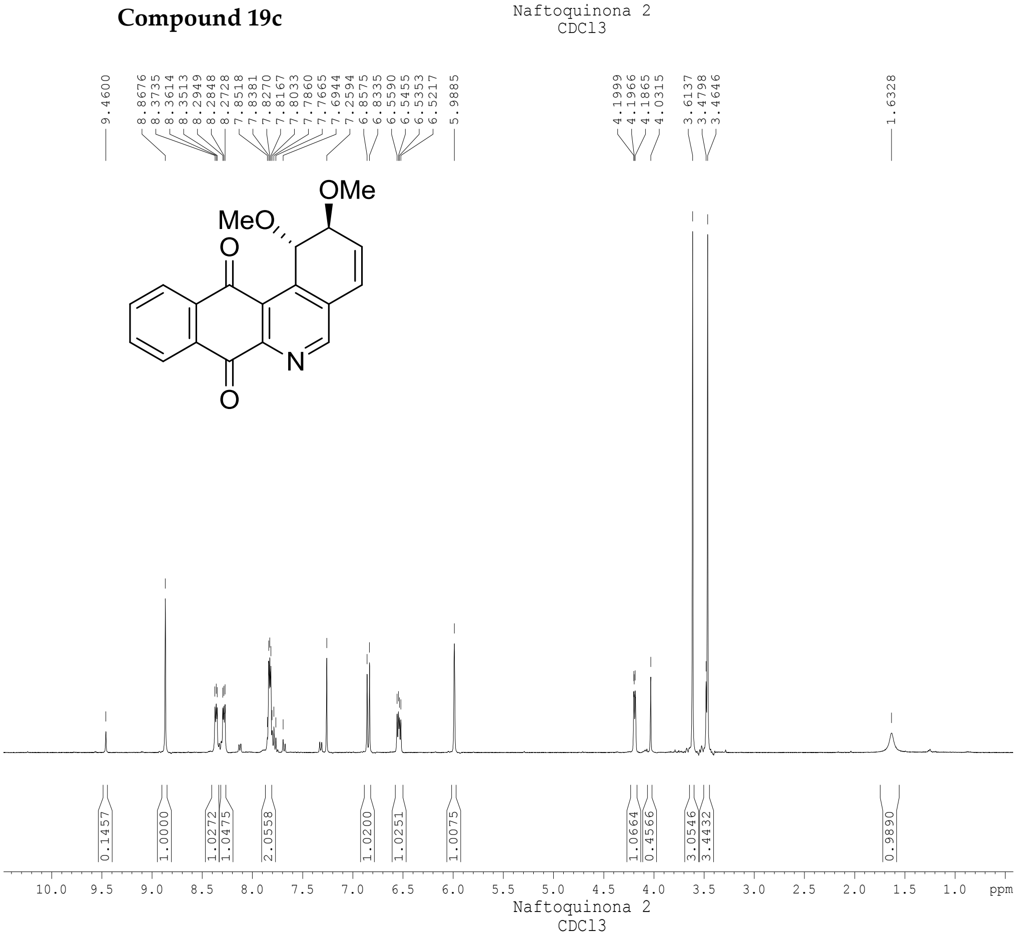

NAME Santiago Hernandez
EXPNO 37
PROCNO 1
Date_ 20151103
Time 12.29
INSTRUM spect
PROBHD 5 mm BBI 1H-BB
PULPROG zg30
TD 32768
SOLVENT CDCl3
NS 8
DS 0
SWH 8012.820 Hz
FIDRES 0.244532 Hz
AQ 2.0447731 sec
RG 1024
DW 62.400 usec
DE 6.00 usec
TE 297.2 K
D1 1.00000000 sec
TD0 1

======== CHANNEL f1 ========
NUC1 1H
P1 6.50 usec
PL1 0.00 dB
SFO1 400.1338012 MHz
SI 16384
SF 400.1300097 MHz
WDW no
SSB 0
LB 0.00 Hz
GB 0
PC 1.00

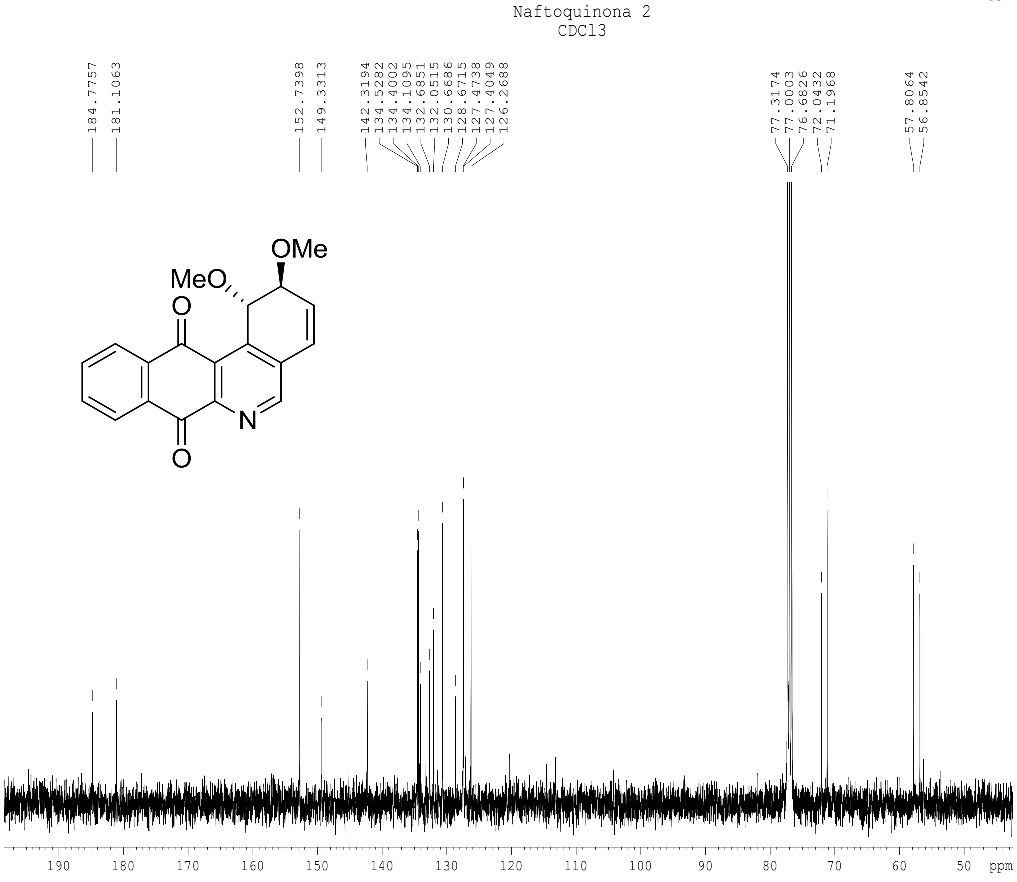

NAME Santiago Hernandez
EXPNO 38
PROCNO 1
Date_ 20151103
Time 12.53
INSTRUM spect
PROBHD 5 mm BBI 1H-BB
PULPROG zgpg30
TD 32768
SOLVENT CDCl3
NS 1536
DS 0
SWH 23980.814 Hz
FIDRES 0.731836 Hz
AQ 0.6832628 sec
RG 574.7
DW 20.850 usec
DE 6.00 usec
TE 297.2 K
D1 2.00000000 sec
D11 0.03000000 sec
TD0 1

======== CHANNEL f1 ========
NUC1 13C
P1 14.00 usec
PL1 -6.00 dB
SFO1 100.6242995 MHz

======== CHANNEL f2 ========
CPDPRG2 waltz16
NUC2 1H
PCPD2 85.00 usec
PL2 6.00 dB
PL12 23.00 dB
PL13 23.00 dB
SFO2 400.1316005 MHz
SI 32768
SF 100.6127709 MHz
WDW EM
SSB 0
LB 1.00 Hz
GB 0
PC 1.40

Compound 20

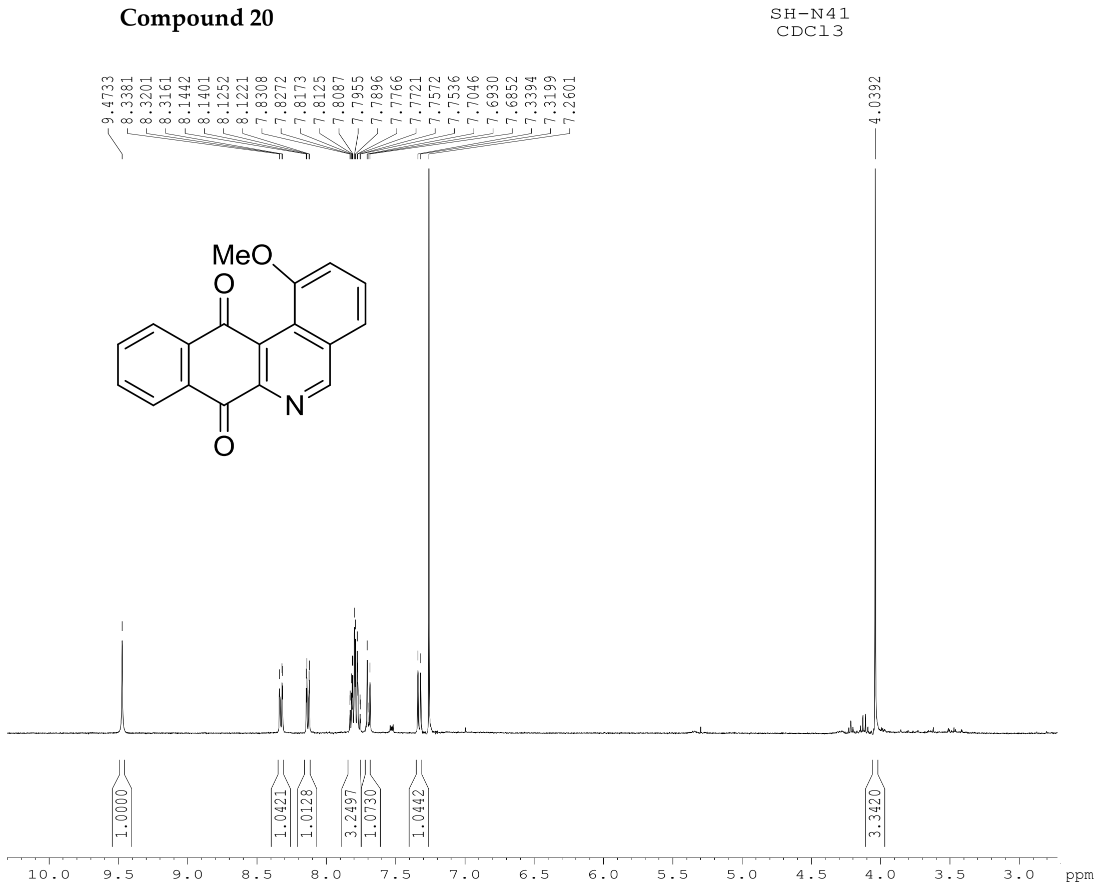

NAME Santiago Hernandez
EXPNO 83
PROCNO 1
Date_ 20160531
Time 15.15
INSTRUM spect
PROBHD 5 mm BBI 1H-BB
PULPROG zg30
TD 32768
SOLVENT CDCl3
NS 8
DS 0
SWH 8012.820 Hz
FIDRES 0.244532 Hz
AQ 2.0447731 sec
RG 812.7
DW 62.400 usec
DE 6.00 usec
TE 295.2 K
D1 1.00000000 sec
TD0 1

======== CHANNEL f1 ========
NUC1 1H
P1 6.50 usec
PL1 0.00 dB
SFO1 400.1338012 MHz
SI 16384
SF 400.1300093 MHz
WDW no
SSB 0
LB 0.00 Hz
GB 0
PC 1.00

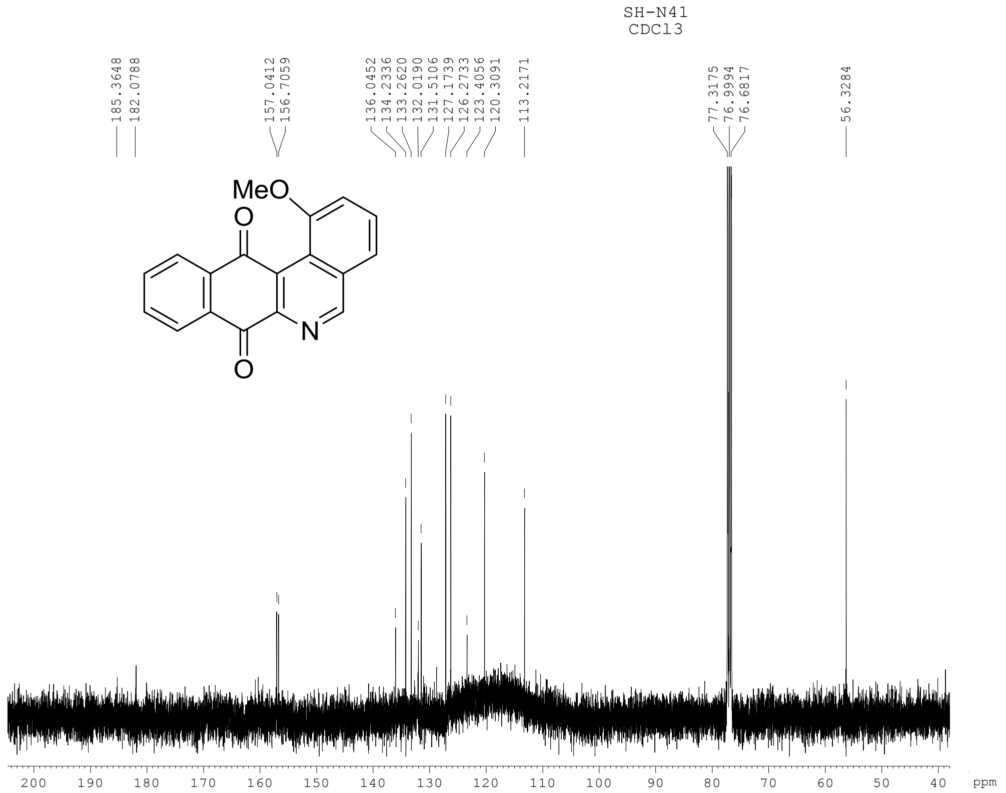

NAME Santiago Hernandez
EXPNO 87
PROCNO 1
Date_ 20160601
Time 6.05
INSTRUM spect
PROBHD 5 mm BBI 1H-BB
PULPROG zgpg30
TD 32768
SOLVENT CDCl3
NS 16000
DS 0
SWH 23980.814 Hz
FIDRES 0.731836 Hz
AQ 0.6832628 sec
RG 645.1
DW 20.850 usec
DE 6.00 usec
TE 297.2 K
D1 2.00000000 sec
D11 0.03000000 sec
TD0 1

======== CHANNEL f1 ========
NUC1 13C
P1 14.00 usec
PL1 -6.00 dB
SFO1 100.6242995 MHz

======== CHANNEL f2 ========
CPDPRG2 waltz16
NUC2 1H
PCPD2 85.00 usec
PL2 6.00 dB
PL12 23.00 dB
PL13 23.00 dB
SFO2 400.1316005 MHz
SI 32768
SF 100.6127699 MHz
WDW no
SSB 0
LB 0.00 Hz
GB 0
PC 1.40

S.2 High resolution mass spectroscopy

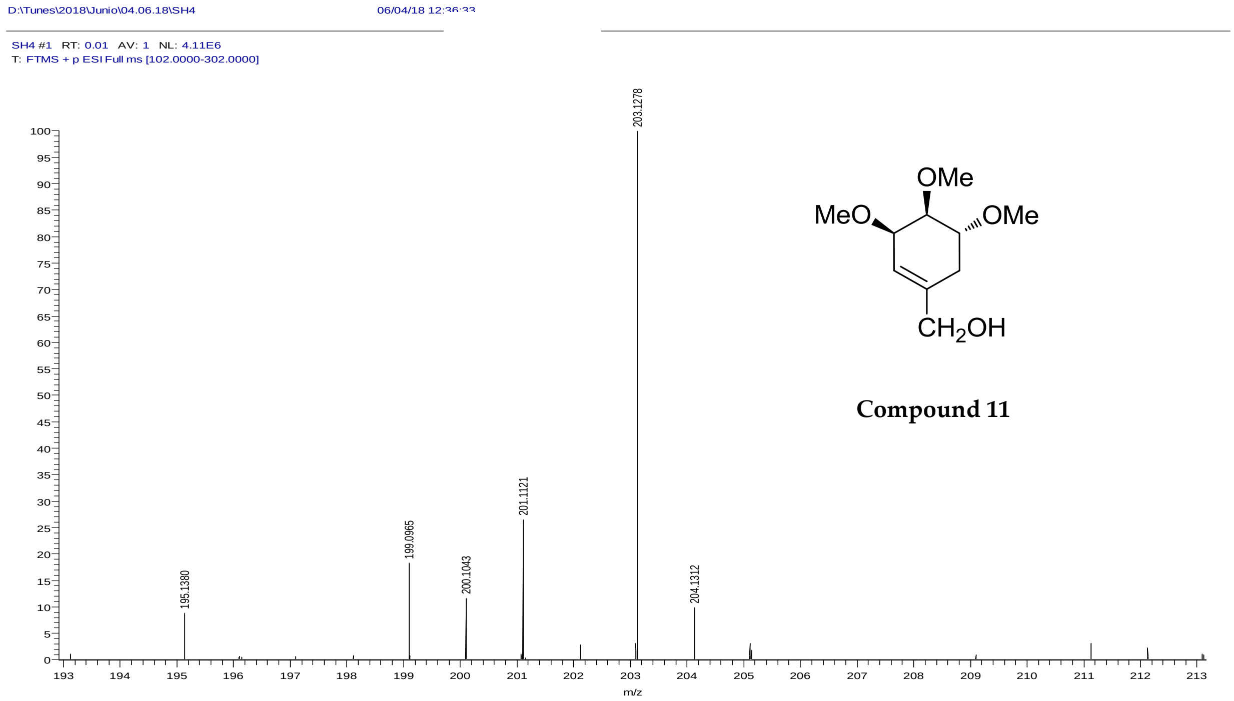

Compound 11

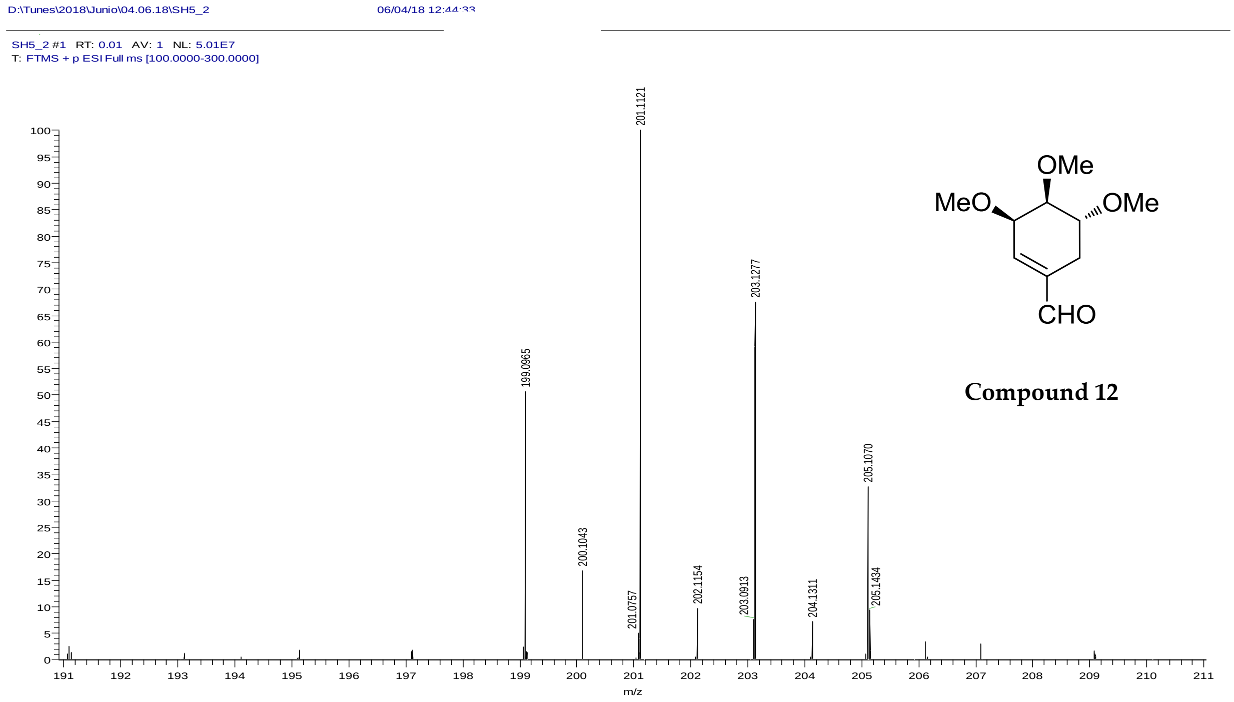

Compound 12

D:\Tunes\2018\Junio\04.06.18\106 06/04/18 12:48:19

106 #1 RT: 0.01 AV: 1 NL: 5.06E8
T: FTMS + p ESI Full ms [143.0000-343.0000]

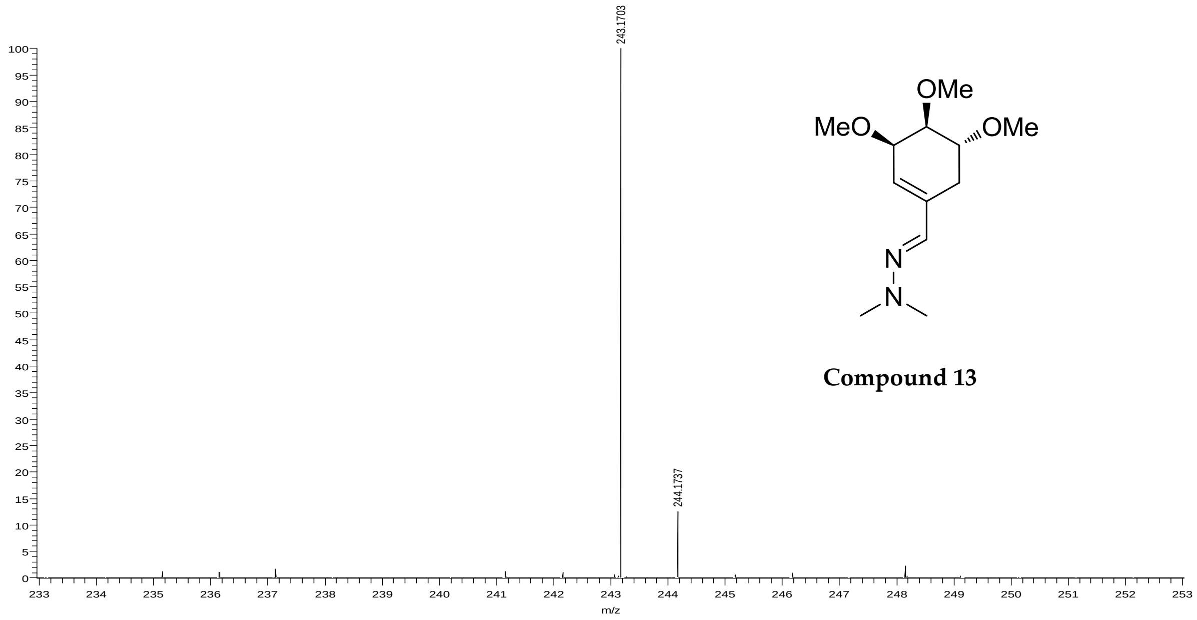

Compound 13

D:\Tunes\2018\Junio\04.06.18\15 06/04/18 12:16:11

15 #1 RT: 0.01 AV: 1 NL: 6.93E6
T: FTMS + p ESI Full ms [298.0000-498.0000]

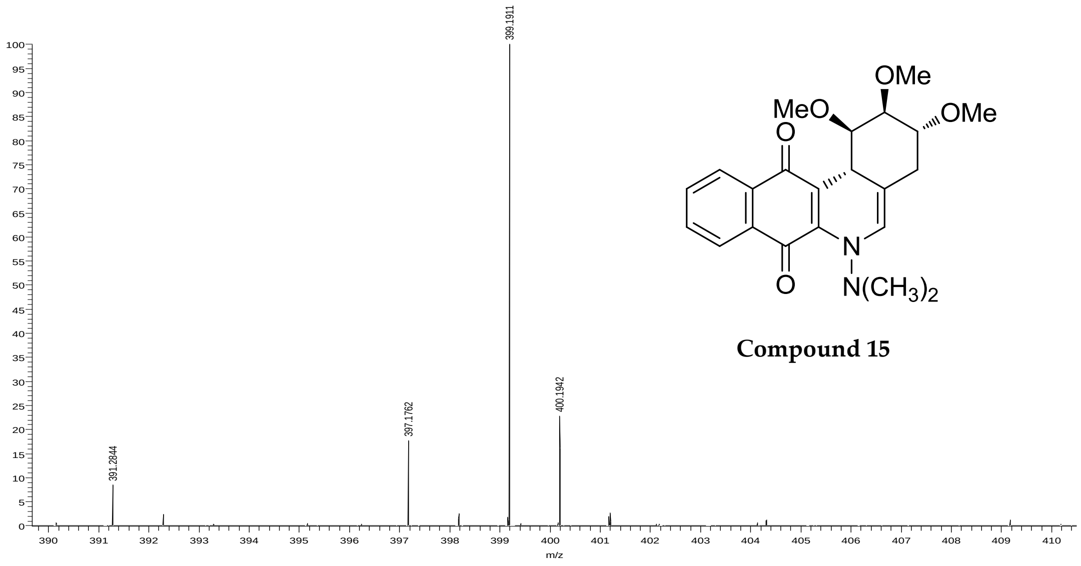

Compound 15

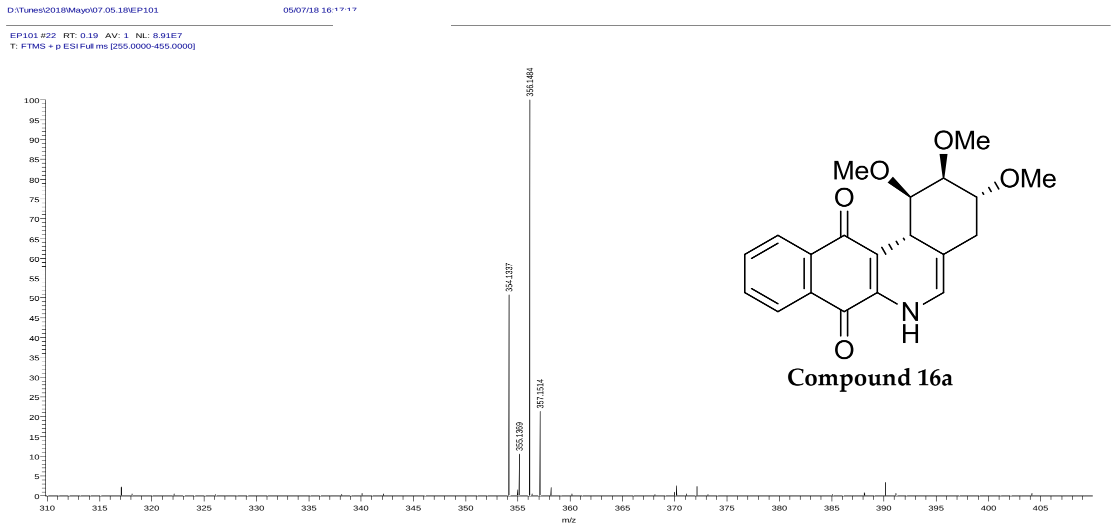
D:\Tunes\2018\Mayo\07.05.18\EP101
05/07/18 16:17:17
EP101 #22 RT: 0.19 AV: 1 NL: 8.91E7
T: FTMS + p ESI Full ms [255.0000-455.0000]
356.1484
354.1337
357.1514
355.1369
m/z
MeO
OMe
OMe
O
O
N
H
Compound 16a

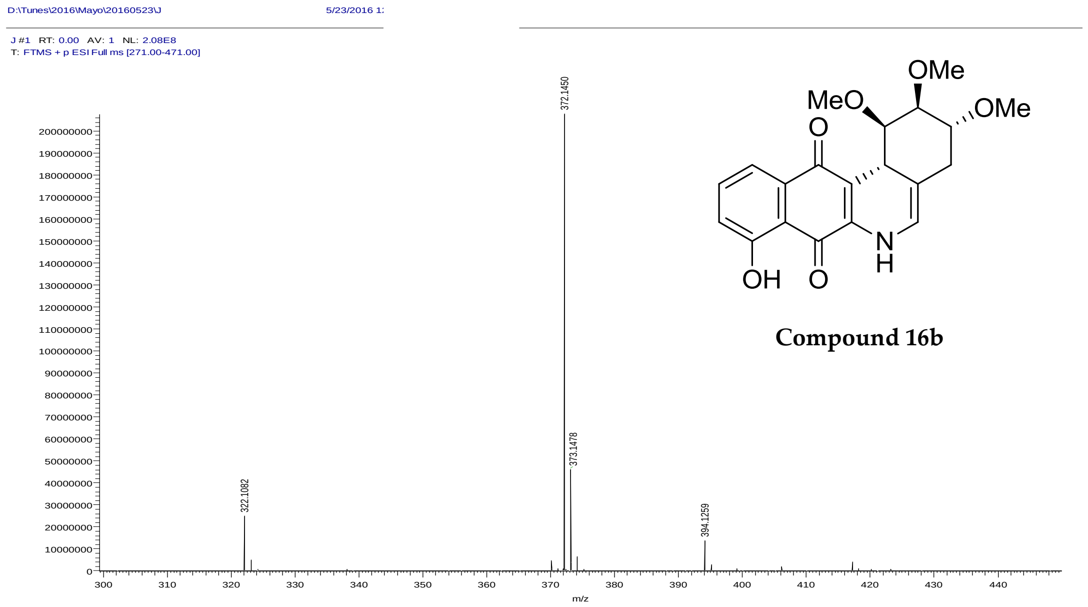
D:\Tunes\2016\Mayo\20160523\J
5/23/2016 1:
J #1 RT: 0.00 AV: 1 NL: 2.08E8
T: FTMS + p ESI Full ms [271.00-471.00]
372.1450
373.1478
322.1082
394.1259
m/z
MeO
OMe
OMe
O
O
OH
N
H
Compound 16b

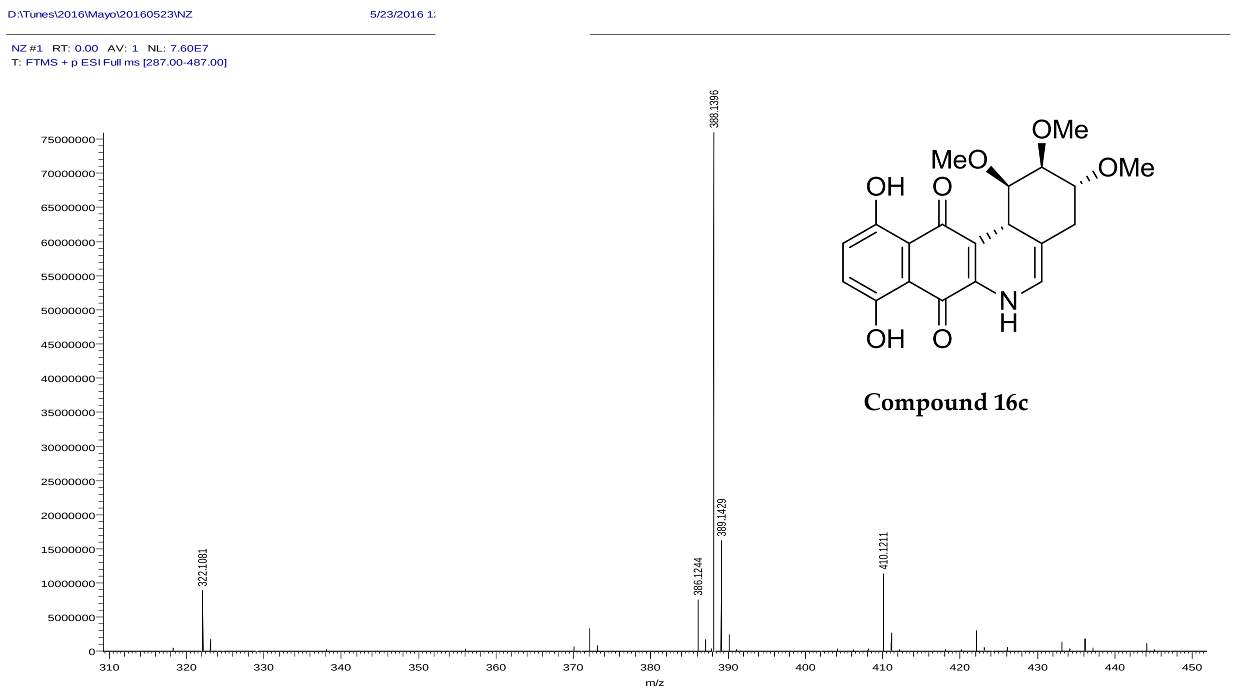
D:\Tunes\2016\Mayo\20160523\NZ
5/23/2016 1:
NZ #1 RT: 0.00 AV: 1 NL: 7.60E7
T: FTMS + p ESI Full ms [287.00-487.00]
388.1396
389.1429
410.1211
386.1244
322.1081
m/z
OMe
MeO
OMe
OH
O
N
H
OH
O
Compound 16c

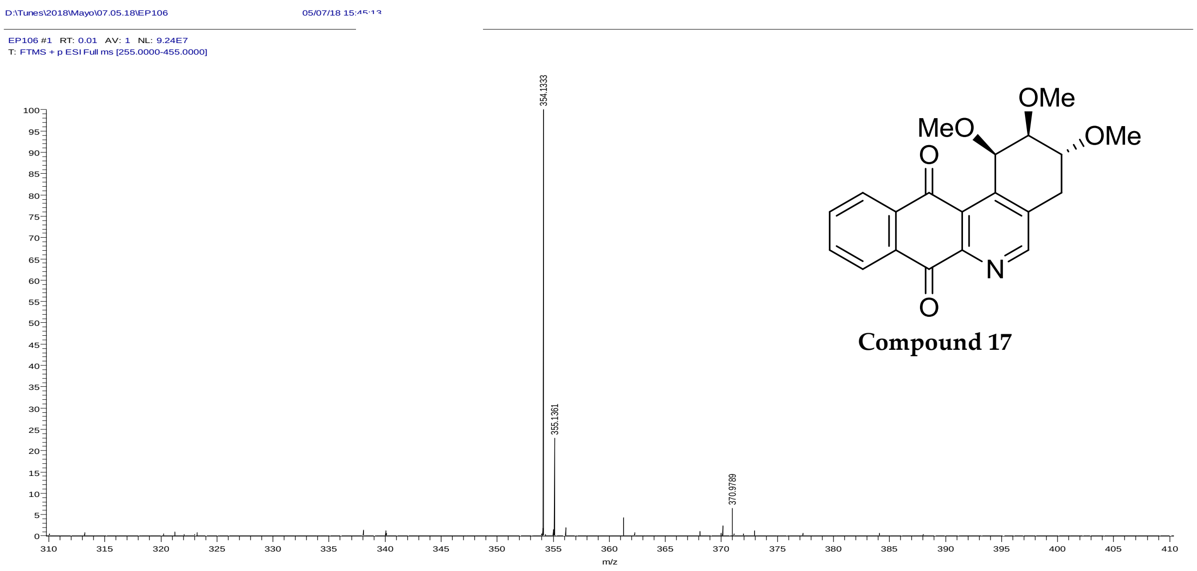
D:\Tunes\2018\Mayo\07.05.18\EP106
EP106 #1 RT: 0.01 AV: 1 NL: 9.24E7
T: FTMS + p ESI Full ms [255.0000-455.0000]
354.1333
355.1361
370.9789
m/z
OMe
MeO
OMe
O
N
O
Compound 17

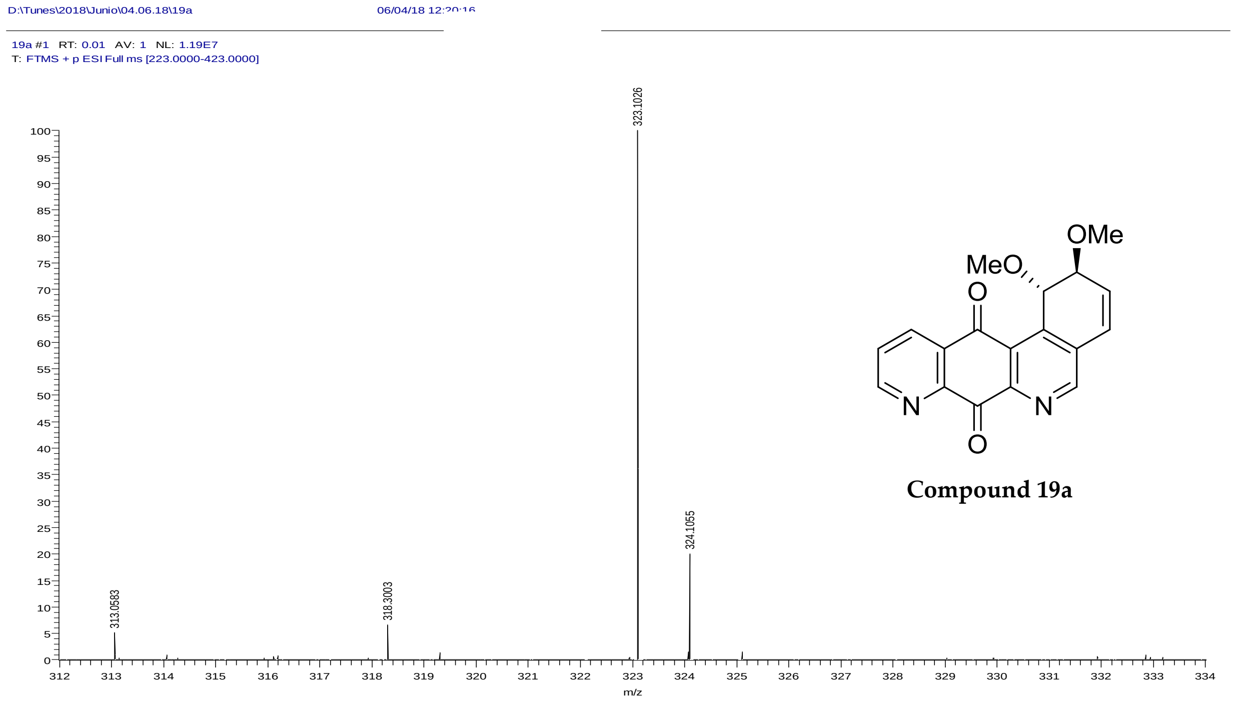
D:\Tunes\2018\Junio\04.06.18\19a
06/04/18 12:20:16
19a #1 RT: 0.01 AV: 1 NL: 1.19E7
T: FTMS + p ESI Full ms [223.0000-423.0000]
323.1026
324.1055
313.0583
318.3003
m/z
OMe
MeO
O
N
N
O
Compound 19a

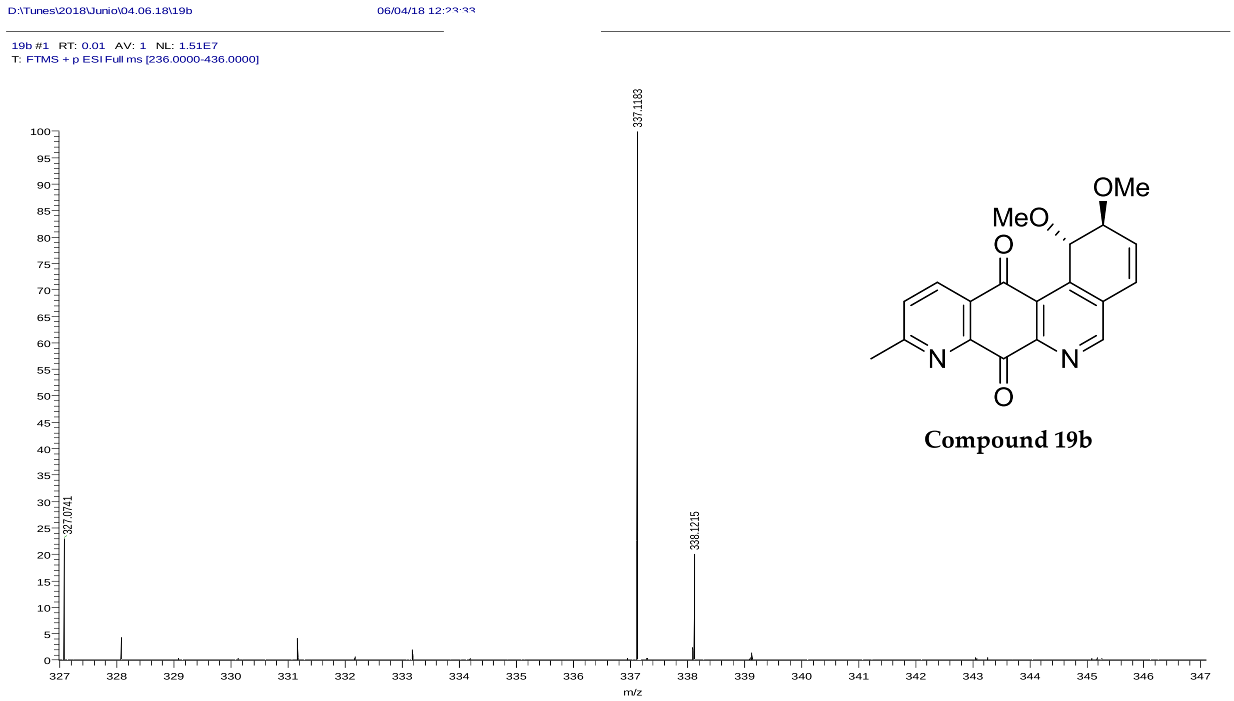
D:\Tunes\2018\Junio\04.06.18\19b
06/04/18 12:23:33
19b #1 RT: 0.01 AV: 1 NL: 1.51E7
T: FTMS + p ESI Full ms [236.0000-436.0000]
337.1183
338.1215
327.0741
m/z
OMe
MeO
O
N
N
O
Compound 19b

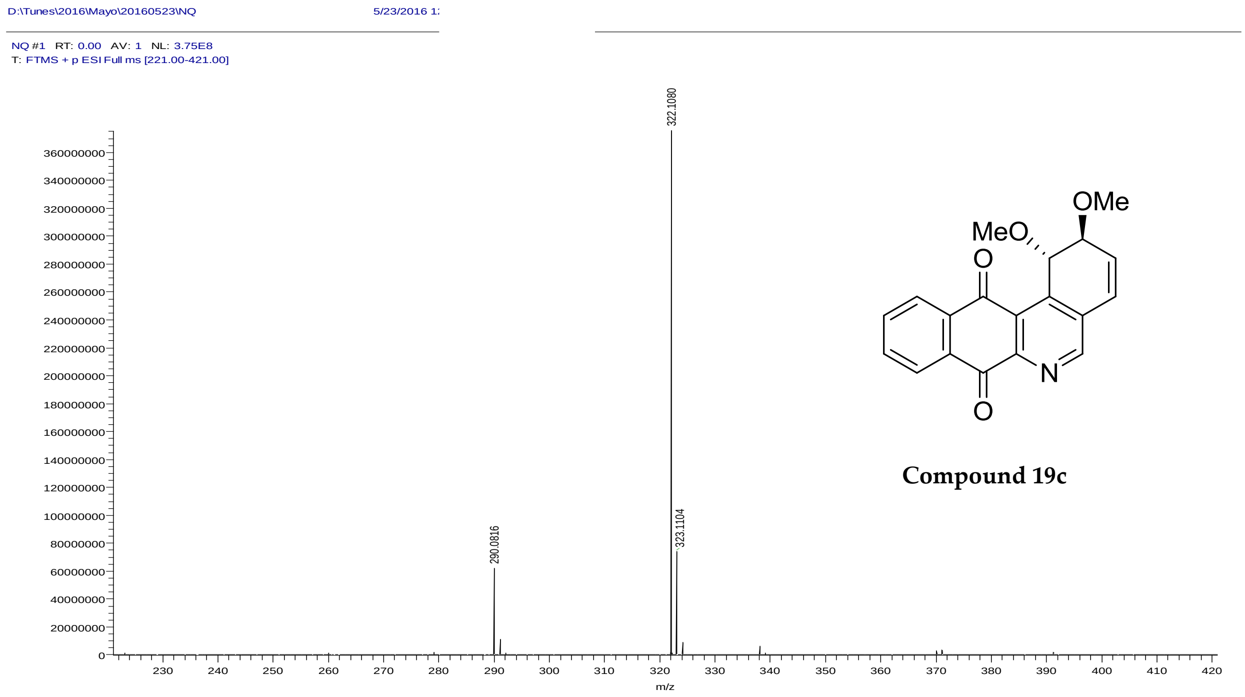

Compound 19c

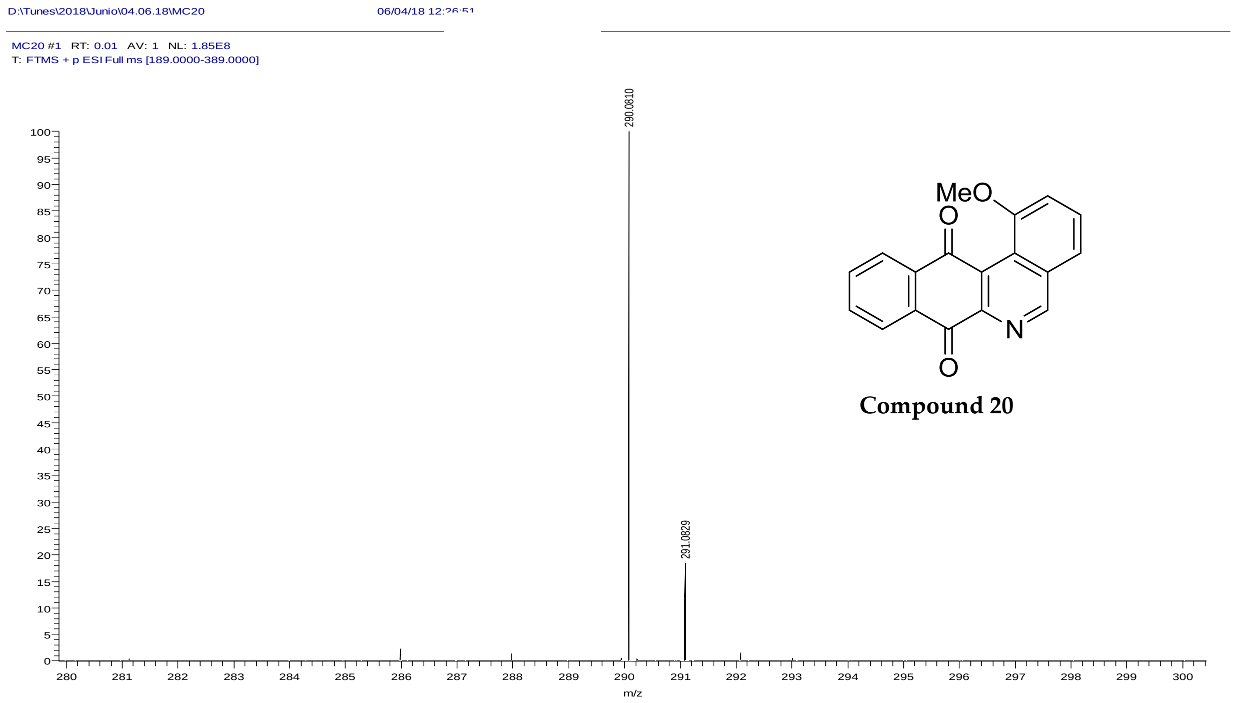

Compound 20

S.3 Infrared spectroscopy

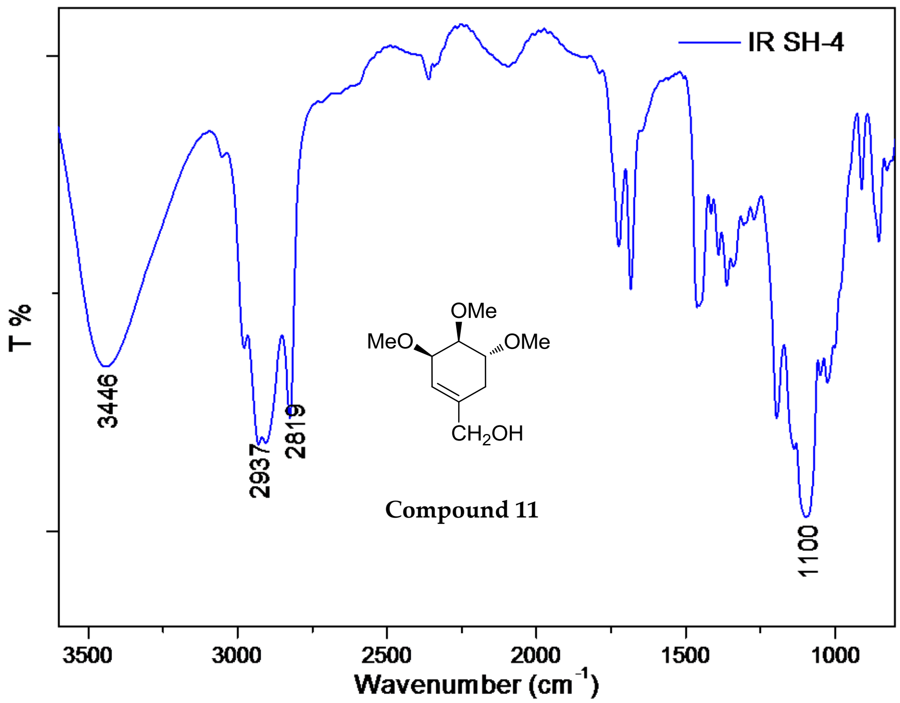

Compound 11

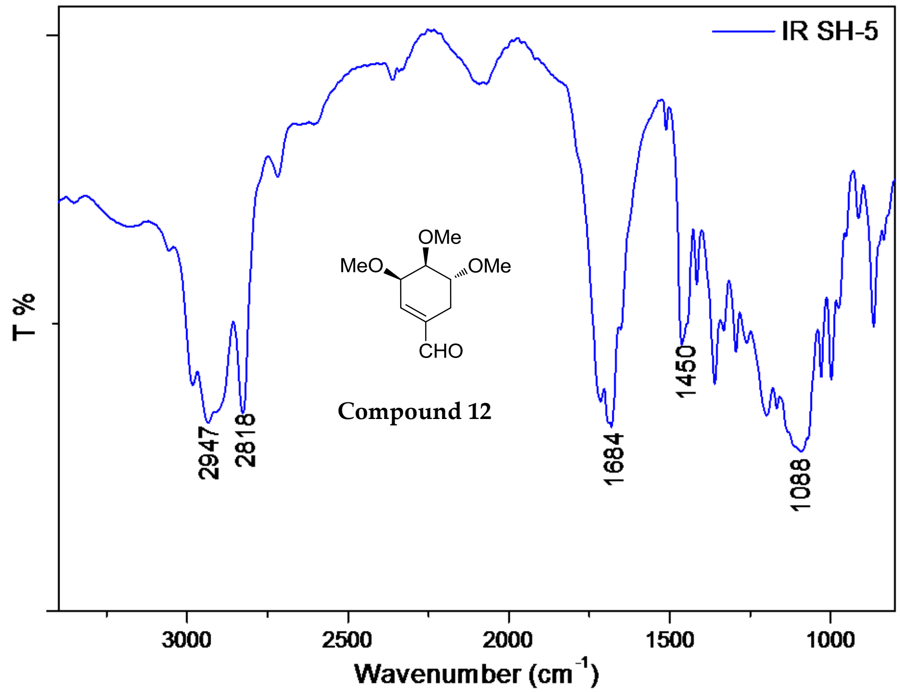

Compound 12

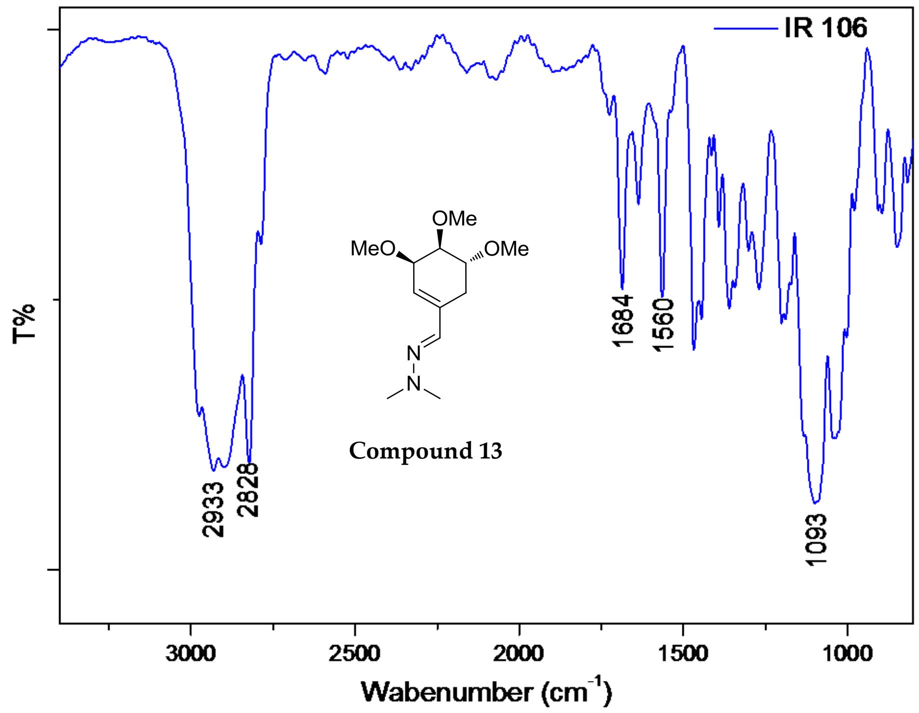
IR 106
T%
OMe
MeO
OMe
N
N
Compound 13
2933
2828
1684
1560
1093
3000
2500
2000
1500
1000
Wabenumber (cm^{-1})

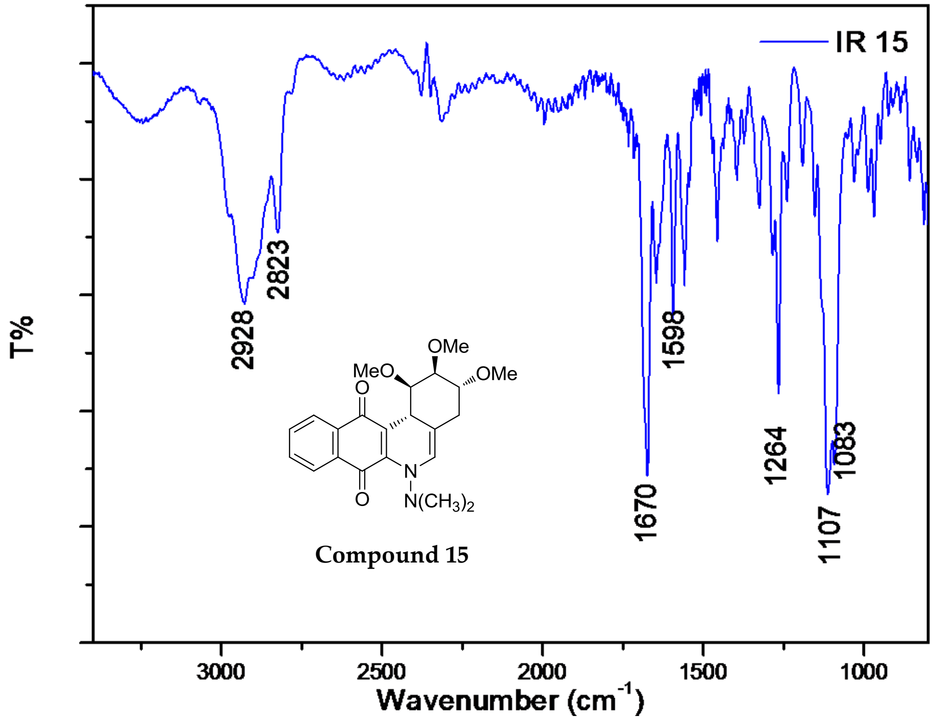
IR 15
T%
OMe
MeO
OMe
O
O
N
N(CH$_3$)$_2$
Compound 15
2928
2823
1670
1598
1264
1107
1083
3000
2500
2000
1500
1000
Wavenumber (cm^{-1})

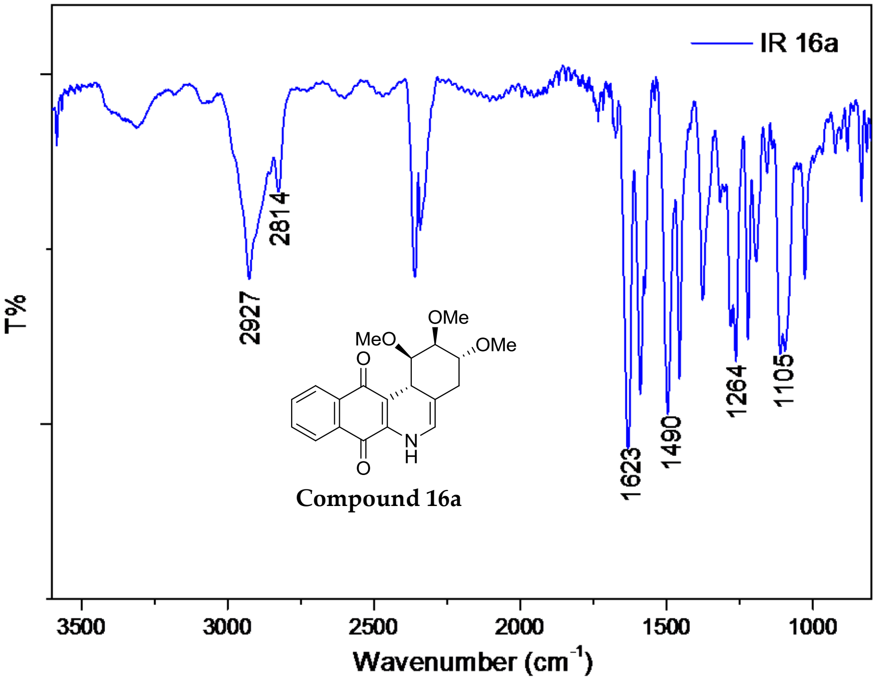

Compound 16a

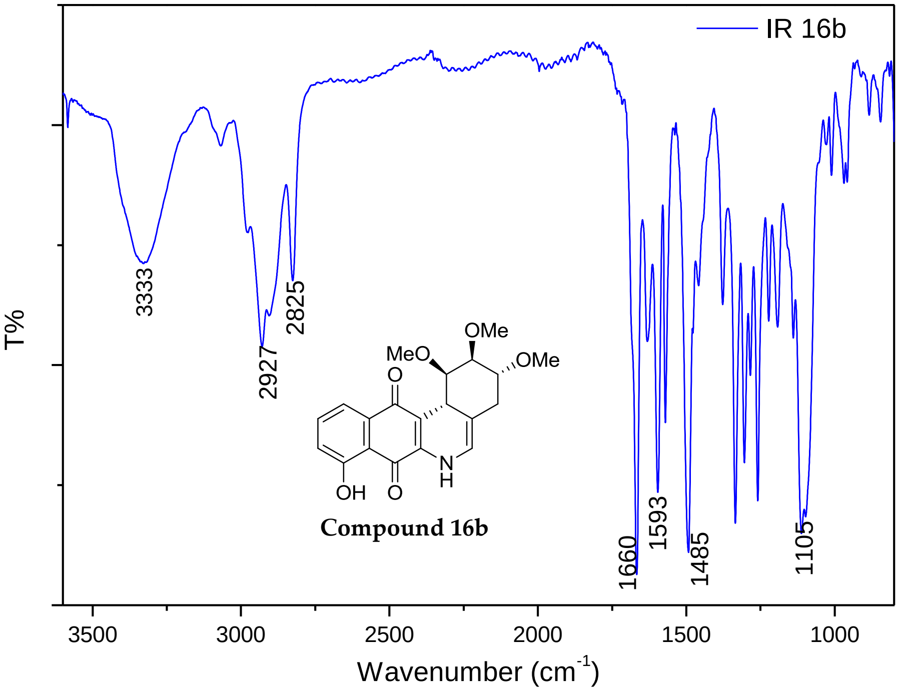

Compound 16b

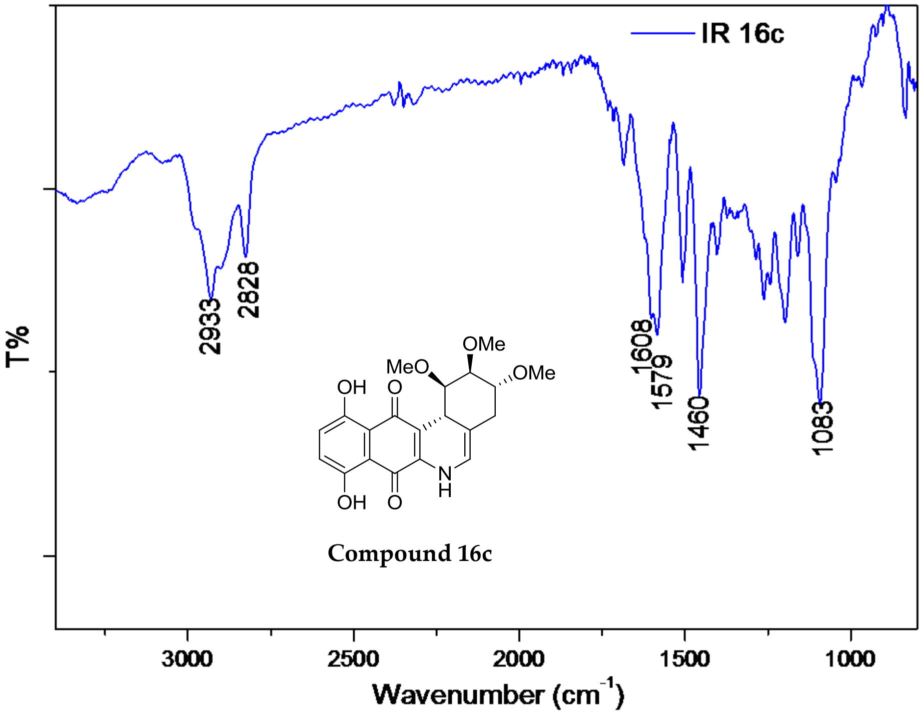

Compound 16c

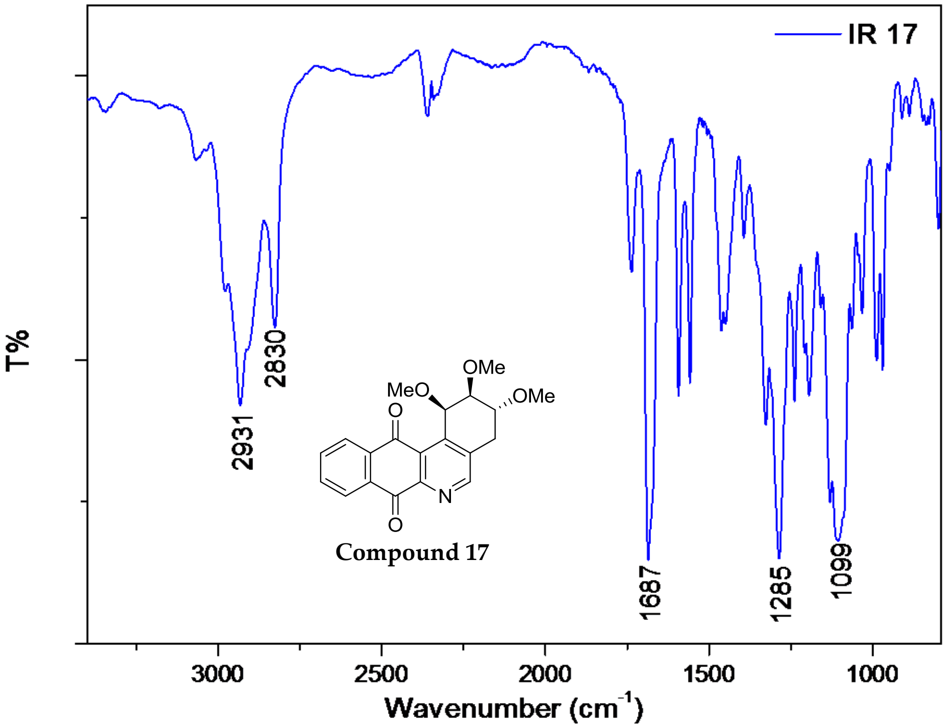

Compound 17

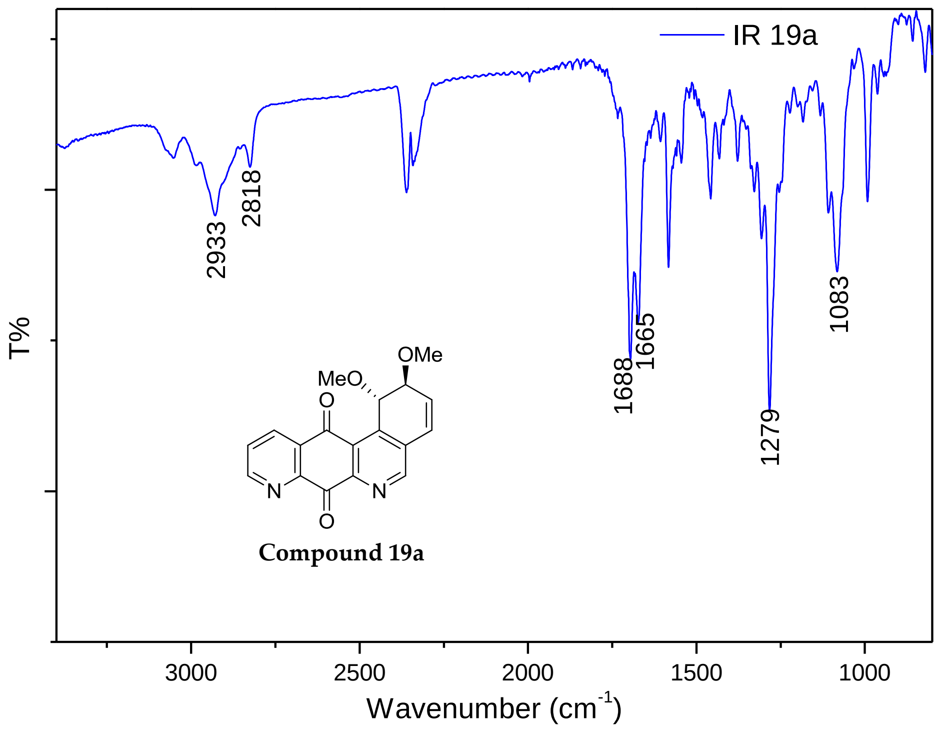
IR 19a
T%
2933
2818
1688
1665
1279
1083
MeO
OMe
O
N
N
O
Compound 19a
3000
2500
2000
1500
1000
Wavenumber (cm-1)

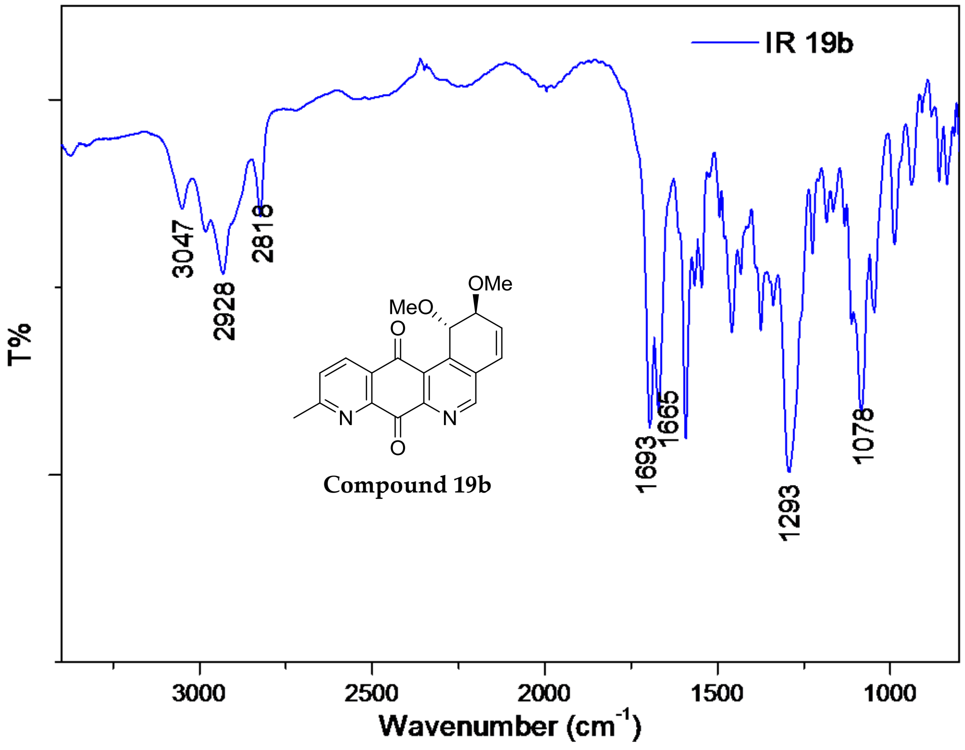
IR 19b
T%
3047
2928
2818
1693
1665
1293
1078
MeO
OMe
O
N
N
O
Compound 19b
3000
2500
2000
1500
1000
Wavenumber (cm-1)

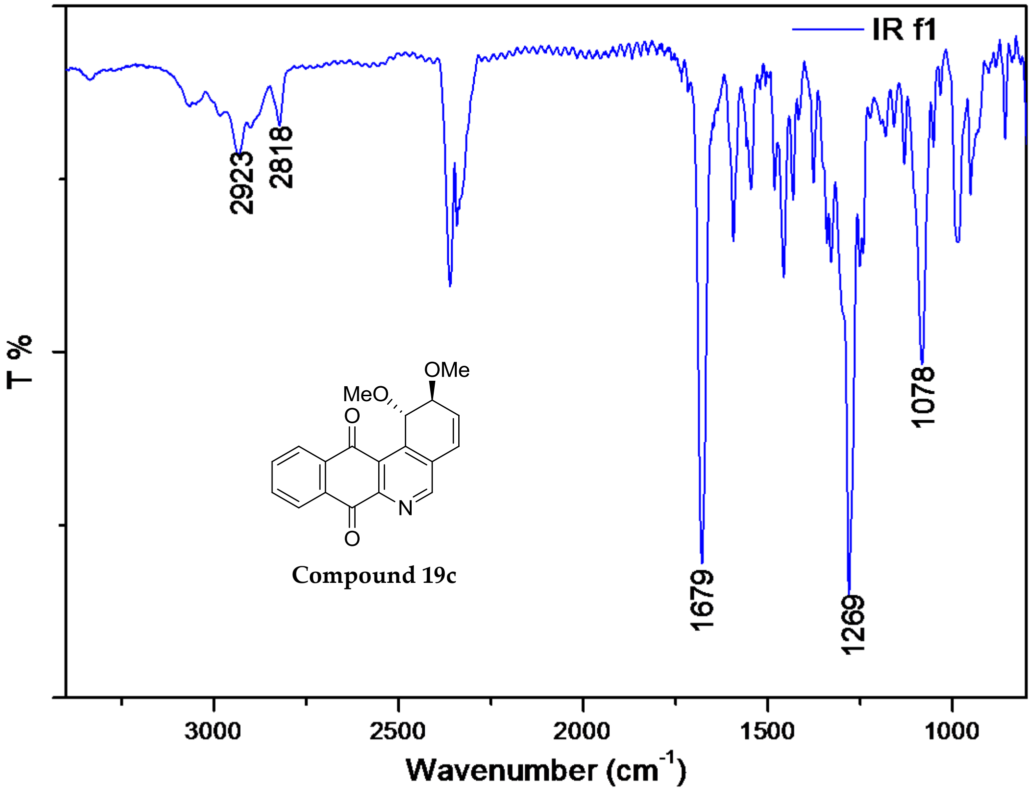
IR f1
T %
2923
2818
1679
1269
1078
MeO
OMe
O
O
N
Compound 19c
3000
2500
2000
1500
1000
Wavenumber (cm-1)

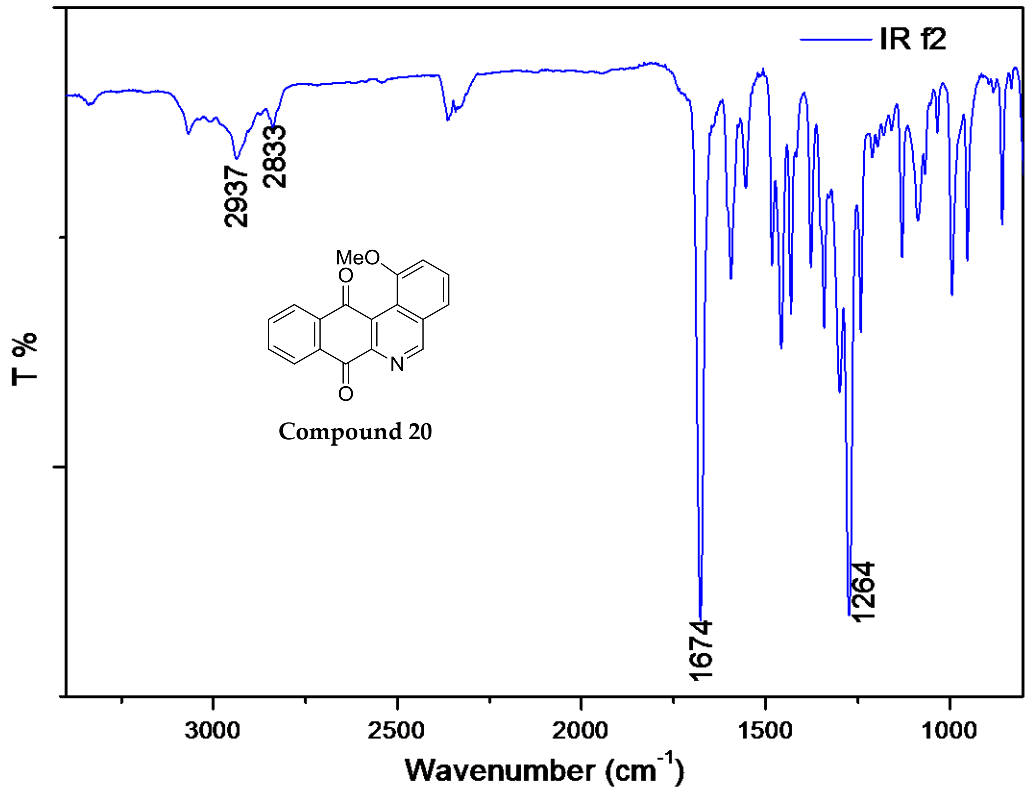
IR f2
T %
2937
2833
1674
1264
MeO
O
O
N
Compound 20
3000
2500
2000
1500
1000
Wavenumber (cm-1)
